# Paleoclimatic modeling and phylogeography of least killifish, *Heterandria formosa*: insights into Pleistocene expansion-contraction dynamics and evolutionary history of North American Coastal Plain freshwater biota

**DOI:** 10.1186/1471-2148-13-223

**Published:** 2013-10-09

**Authors:** Justin C Bagley, Michael Sandel, Joseph Travis, María de Lourdes Lozano-Vilano, Jerald B Johnson

**Affiliations:** 1 Department of Biology, Brigham Young University, 401 WIDB, Provo, UT 84602, USA; 2 Monte L. Bean Life Science Museum, Brigham Young University, Provo, UT 84602, USA; 3 Department of Biological Science, Biodiversity & Systematics, The University of Alabama, Box 870345, Tuscaloosa, AL 35487, USA; 4 Department of Biological Science, The Florida State University, Tallahassee, FL 32306, USA; 5 Laboratorio de Ictiología, Facultad de Ciencias Biológicas, Universidad Autónoma de Nuevo León, Monterrey, Nuevo León, México

## Abstract

**Background:**

Climatic and sea-level fluctuations throughout the last Pleistocene glacial cycle (~130-0 ka) profoundly influenced present-day distributions and genetic diversity of Northern Hemisphere biotas by forcing range contractions in many species during the glacial advance and allowing expansion following glacial retreat ('expansion-contraction’ model). Evidence for such range dynamics and refugia in the unglaciated Gulf-Atlantic Coastal Plain stems largely from terrestrial species, and aquatic species Pleistocene responses remain relatively uninvestigated. *Heterandria formosa*, a wide-ranging regional endemic, presents an ideal system to test the expansion-contraction model within this biota. By integrating ecological niche modeling and phylogeography, we infer the Pleistocene history of this livebearing fish (Poeciliidae) and test for several predicted distributional and genetic effects of the last glaciation.

**Results:**

Paleoclimatic models predicted range contraction to a single southwest Florida peninsula refugium during the Last Glacial Maximum, followed by northward expansion. We inferred spatial-population subdivision into four groups that reflect genetic barriers outside this refuge. Several other features of the genetic data were consistent with predictions derived from an expansion-contraction model: limited intraspecific divergence (e.g. mean mtDNA p-distance = 0.66%); a pattern of mtDNA diversity (mean *Hd* = 0.934; mean π = 0.007) consistent with rapid, recent population expansion; a lack of mtDNA isolation-by-distance; and clinal variation in allozyme diversity with higher diversity at lower latitudes near the predicted refugium. Statistical tests of mismatch distributions and coalescent simulations of the gene tree lent greater support to a scenario of post-glacial expansion and diversification from a single refugium than to any other model examined (e.g. multiple-refugia scenarios).

**Conclusions:**

Congruent results from diverse data indicate *H. formosa* fits the classic Pleistocene expansion-contraction model, even as the genetic data suggest additional ecological influences on population structure. While evidence for Plio-Pleistocene Gulf Coast vicariance is well described for many freshwater species presently codistributed with *H. formosa*, this species demography and diversification departs notably from this pattern. Species-specific expansion-contraction dynamics may therefore have figured more prominently in shaping Coastal Plain evolutionary history than previously thought. Our findings bolster growing appreciation for the complexity of phylogeographical structuring within North America’s southern refugia, including responses of Coastal Plain freshwater biota to Pleistocene climatic fluctuations.

## Background

Present-day distributions of many of Earth’s biotas reflect the profound influence of climatic and sea-level fluctuations during the glacial-interglacial cycles of the Pleistocene (2.58-0.01 million years ago, Ma; [1]) [2-4]. Notably, eight glacial advances since 740 ka have produced more extreme 100,000-year environmental changes than those preceding 1 Ma [5]. Because glacial-interglacial transitions occurred in an evolutionary blink of an eye, many extant species have spent most of their recent evolutionary histories under glacial rather than short-lived (~10,000-20,000 years) interglacial conditions [6]. Therefore, extreme environmental changes associated with the Last Glacial Maximum (LGM; ~22-19 ka) may have been the most recent climatic factors that affected present-day patterns of biodiversity prior to the global proliferation of humans and anthropogenic environmental disturbance [2,6].

The biogeographical consequences of Pleistocene glaciations are generally thought of in terms of direct effects of glacial cover and temperature change on Northern Hemisphere biotas. This is because over 80% of glacial ice on Earth resided in the Northern Hemisphere during the LGM, resulting in the destruction and reorganization of continental landscapes in both the Old and New World e.g. [2,7]. While few species from glaciated areas persisted *in situ* by adapting to changing local environments, many were actively displaced (tracking suitable habitat), passively displaced (local extinction, regional extirpation), or went extinct [3,8]. Indeed, abundant paleontological and palynological evidence indicates that many terrestrial species of North America and Europe contracted their ranges during the LGM to one or more lower-latitude (or altitude) refugia, then expanded northward to recolonize much of their present-day ranges during the warmer Holocene interglacial [9-14]. This scenario is known as the 'expansion-contraction’ model of Pleistocene biogeography and has reached paradigm status in biology [15].

Many patterns of genetic variation are consistent with predictions from a model of range contraction during glacial advance and rapid post-glacial expansion. For example, phylogeographical analyses across European flora and fauna have identified the Iberian, Italian and Balkan peninsulas as three main southern refugia from which postglacial recolonization of northern Europe took place [9,16,17]. In more recent work, the tools of phylogeography and ecological niche modeling e.g. [18] have been joined to refine models of Pleistocene expansion and contraction. Such analyses have revealed complicated patterns of northern and southern LGM refugia and complex histories of speciation, dispersal, secondary contact, and demographic expansion of species within the southern refugia themselves, particularly in Europe [15,18-24].

**Figure 1:**
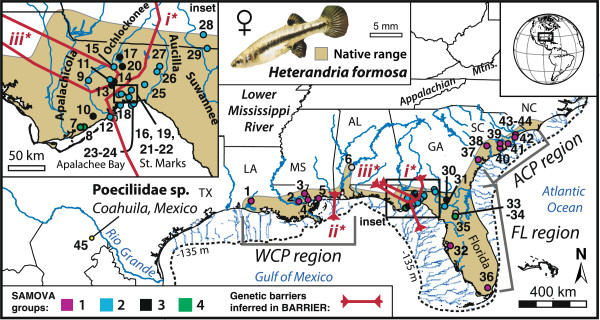
***Heterandria formosa *modern, native geographical distribution and collection sites.** Sites (dots) correspond to exact localities and sample sizes in Table 1. Brackets indicate regional groups discussed in the text and used in analyses (WCP, Western Coastal Plain; FL, Florida; ACP, Atlantic Coastal Plain). Upper left inset shows a close-up of Apalachicola River/Bay and Apalachee Bay zones and hydrography. Rivers are blue lines; predicted river paths to the Pleistocene LGM coastline (dotted line) are given based on GIS-based bathymetric modeling at -110 m (provided by P. J. Unmack).

There is substantial geoscientific evidence that the glacial-interglacial cycles also had substantial effects in subtropical and tropical zones, including refugial areas recognized today that were not covered by glaciers [7,19-21]. During the Last Interglaciation (LIG; 130-116 ka; including 'Sangamonian’ stage, ~125 ka), wetter, warmer climate and sea levels ~4-6 m above present sea level (ASL) [25,26] created favorable conditions for expansions in many species ranges. Subsequent global cooling (~4-10°C cooler surface air temperatures [27]) and aridification during the LGM caused eustatic sea levels to drop ~120-130 m lower than today [28], exposing huge areas of continental shelf, areas large enough to have doubled the span of the Florida peninsula to 600 km wide. For freshwater species, this increase in LGM land area also increased river lengths (Figure 1), which likely increased population connectivity through river anastomoses. Such mixing of populations from formerly isolated drainages would have promoted dispersal and gene flow, and may have aided southward range shifts. Thus while glacial stages are perceived as periods of reduced population sizes and isolation in refugia [9], for some species, particularly those in fresh waters, they may have presented opportunities for coastal dispersal, leading to range expansion or shifting (contraction). The possibility for southern refugia to act as areas of long-term persistence, dispersal, and gene flow, rather than range expansion-contraction dynamics, is known as the 'southern crossroads’ hypothesis [21].

The Gulf-Atlantic Coastal Plain of eastern North America presents an ideal system in which to study interactions between geology, climate and diversification of a unique biota. This unglaciated region has figured prominently in phylogeographic research, and has revealed several major genetic breaks and confirmed local glacial refugia for northern taxa and endemic coastal species [29,30]. Many Coastal Plain species possess evolutionary lineages that have diverged in an east-to-west direction across common biogeographic barriers, including peninsular Florida which separates the maritime Atlantic-vs.-Gulf coast biota, the Apalachicola River (Gulf-Atlantic slope drainages), Mobile Bay, and the Mississippi River [30-32]. Genetically identified breaks in this region also coincide with species distributional breaks [33] and thus likely reflect historical vicariance events, or recurrent vicariance coupled with extinction-recolonization of populations [29,31,34]. However, phylogeographical studies also indicate that species persisted through the LGM in refugia on either side of the Apalachicola basin in Florida and Texas-western Louisiana [29,31]. Between these regions, there are north-south-trending zones of hybridization and secondary contact, including one of Remington’s 'suture zones’ between Alabama and northwest Florida, indicating a meeting place for lineages undergoing northward postglacial expansion [30,35]. As is the case for the patterns in evidence for expansion-contraction dynamics in unglaciated regions of Europe [2,4,9], evidence for these refugia in North America has been developed mainly from terrestrial taxa, e.g. mints (e.g. *Conradina*[36,37]) and yellow-poplar (*Liriodendron tulipifera*[38]). These terrestrial data suggest that other codistributed species may have experienced similar range dynamics fitting the expansion-contraction model, possibly involving similar refugia. However, while regional genetic breaks are well documented, Pleistocene evolutionary responses of Coastal Plain aquatic biota remain relatively uninvestigated.

Here, we focus on least killifish, *Heterandria formosa* Girard 1859, a species which presents several advantages as a historical biogeographic study system that likely exhibited distinct response(s) to the climatic upheavals of the Late Pleistocene. First, these small (12-30 mm) livebearing freshwater fish (family Poeciliidae) are restricted to low-elevation Coastal Plain areas of subtropical humid climate zones (hot, wet summers and mild winters), and the tropical Everglades [39]. This suggests climate is likely an important factor limiting *H. formosa* distribution. This species is also intolerant to cold-seasonal temperatures, making it an ideal candidate for ecological niche modeling and for testing the expansion-contraction model. Second, *H. formosa* span the Florida peninsula, an important glacial-stage refugium [29,35]; thus, it is plausible that populations were displaced to warmer south-peninsula areas during the LGM. Third, *H. formosa* display ecological characteristics consistent with the potential for rapid population expansion over evolutionary timescales, including (i) rapid reproduction and short generation time (*T* ≈ 0.33 yr [40]); (ii) a range of tolerances to different abiotic conditions [39,40], and (iii) female capacity for livebearing and sperm storage, suggesting individuals or groups of migrating females could found populations during rapid post-LGM expansions [41]. Fourth, *H. formosa* is endemic to the Gulf-Atlantic Coastal Plain and thus also offer the opportunity to test the generality of the standard regional vicariance model and phylogeographical breaks documented in other taxa.

A previous rangewide study by Baer [42] using allozymes inferred genetic barriers in *H. formosa* between the Western Coastal Plain (WCP) and Atlantic Coastal Plain (ACP) regions (Figure 1), around the Suwannee River, but not between north Florida and the ACP. Another break occurred between Gulf-draining Waccasassa and Withlacoochee Rivers, creating a clade of Louisiana plus south Florida samples. Baer [42] also hypothesized an important role for population expansion and cross-peninsula gene flow in influencing current levels of genetic variation—including recent founding of ACP populations after Early Pleistocene high seas (≤1.5 Ma; 'northeast colonization’ hypothesis), supported by lower genetic diversity and a lack of isolation-by-distance only among ACP populations. Yet while Baer’s [42] study presents a broad outline for understanding *H. formosa* historical biogeography, the limited levels of genetic variation he found and the more modest methods available at the time limited the hypotheses that could be tested. The current analytical framework in historical biogeography, including our ability to combine ecological niche modeling (e.g. to infer Pleistocene-Holocene distributions and generate spatially explicit hypotheses) with coalescent simulations (e.g. used to discriminate among historical scenarios [43,44]), provides a more rigorous framework for biogeographical inference in this species [45-47].

In this study, we integrate ecological niche modeling with paleoclimatic data, phylogeography, and coalescent simulations to infer the historical biogeography of *H. formosa*. Specifically, we use *H. formosa* to test the hypothesis that members of the Coastal Plain freshwater biota fit predictions of the expansion-contraction model (as opposed to southern crossroads, or regional vicariance and northeast colonization hypotheses) by testing for predicted patterns of Late Pleistocene range shifts, refugia and recolonization patterns and their spatial-genetic effects. In addition to analyzing new mtDNA and nDNA sequences, we re-analyze available allozyme data [42], which provide a novel temporal spectrum and improve our tests of genetic diversity predictions, including latitudinal patterns not previously investigated. We show that these new data, combined with paleoclimatic modeling in an integrative approach, support a hypothesis of Pleistocene range expansion-contraction dynamics in least killifish.

## Methods

### Sampling and laboratory methods

Rather than exhaustively sampling every *H. formosa* population rangewide, we conducted higher-density sampling near the middle of the range in northern Florida to get at finer-scale patterns of diversity and gene flow near the area where Baer [42] predicted the main genetic breaks to occur. We also sampled broadly to infer phylogeographical patterns and test rangewide models of population history. We collected 224 *H. formosa* individuals from 42 coastal sites across the species range (1-12 individuals/site; Figure 1; Table 1), which we assumed represented discrete populations. Specimens were collected using standard seining and electrofishing techniques, preserved in 95% ethanol, transported to the laboratory, and maintained at room temperature before DNA extraction. Studies of population mutation rate parameter *θ* demonstrate that N ≈ 8 samples are typically sufficient for characterizing genetic diversity, though estimates are improved by recombination and more loci [48,49]. Thus, wherever possible during our analyses, we used samples that met this N ≥ 8 threshold-sampling criterion, or pooled samples across populations until this threshold was met. We included three samples of a novel taxon (hereafter, 'Poeciliidae sp.’) collected from Coahuila, Mexico in our analyses. Based on morphological variation we have uncovered while describing this recently discovered taxon, we hypothesize it is the lineage sister to *H. formosa* (MLLV, JCB, and JBJ, unpublished data). We tested this hypothesis through outgroup analysis by adding 72 sequences from 23 other poeciliid lineages retrieved from GenBank into our phylogenetic alignments [*Belonesox belizanus*; *Gambusia affinis*; *Limia dominicensis*, *L. melanogaster*, *L. tridens*, and *L. vittata*; *Pamphorichthys hollandi*; *Pseudoxiphophorus* (15 lineages); and *Xiphophorus helleri*; Additional file 1: Table S1]. This yielded a total of 24 possible outgroup lineages.

**Table 1:** ***Heterandria formosa *collection sites, sample sizes and genetic diversity measures**

**Population (specimen no. code†)**	**No.**	**Locality**	**Latitude (° N)**	**Longitude (° W)**	**N**	**Cyt*b *haps.**	**Study region**
**Bayou Nezpique (BNE *x *LA)**	1	Bayou Nezpique, south of I-10, ~30 km east of Lake Charles, Cameron Par., LA	30.245	-92.627	2	22	WCP
**Lake Pontchartrain (PON *x *LA)**	2	Lake Pontchartrain, LA	30.189	-90.101	1	31	WCP
**Bogue Chitto (BOG *x *LA)**	3	Bogue Chitto R. overflow pool along LA Hwy 21 south of Sun, St. Tammany Par., LA	30.630	-89.897	2	30	WCP
**Porter's River (POR *x *LA)**	4	Porter's R. at Davis Landing Rd., St. Tammany Par., LA	30.318	-89.728	2	40, 41	WCP
**Wolf Creek Swamp (WCMS *x *)**	5	Wolf Cr. swamp at Belle Ferry Rd., Harrison Co., MS	30.387	-89.197	2	42	WCP
**Alabama River trib. (ALA *x *AL)**	6	Unnamed trib. to Alabama R. (Bailey Cr. origin), Wilcox Co., AL	31.981	-87.504	2	‒	‒
**Five Points (FIV *x *FL)**	7	Unnamed trib. at Five Points, Tate's Hell State Forest, Franklin Co., FL	29.893	-84.760	9	17, 29	FL
**Crooked River (CRO *x *FL)**	8	Crooked R. at Tate's Hell State Forest, Franklin Co., FL	29.893	-84.731	10	17	FL
**Ochlockonee River (OCH *x *FL)**	9	Ochlockonee R. at Blountstown Hwy, Leon Co., FL	30.388	-84.665	2	1, 37	FL
**Womack Creek (WOM *x *FL)**	10	Womack Cr. at Apalachicola National Forest, Franklin Co., FL	30.009	-84.608	9	1, 3, 11-14	FL
**Moore Lake (MOO *x *FL)**	11	Moore Lake at Apalachicola National Forest, Leon Co., FL	30.392	-84.407	10	24	FL
**Hill Swale (HIL *x *FL)**	12	Hill Swale, just north of Ochlockonee Bay, Wakulla Co., FL	29.100	-84.406	10	25, 26	FL
**Trout Pond (TRO *x *FL)**	13	Trout Pond at Apalachicola National Forest, Leon Co., FL	30.335	-84.385	10	3	FL
**Cessna Pond (CES *x *FL)**	14	Cessna Pond at Apalachicola National Forest, Leon Co., FL	30.374	-84.360	9	3, 28	FL
**Little Lake (LIT *x *FL)**	15	Little Lake Jackson, west of Hwy 27, northwest Tallahassee, Leon Co., FL	30.526	-84.359	6	2	FL
**Wakulla Springs (WAK *x *FL)**	16	Wakulla Springs at Edward Ball Wakulla Springs State Park, Wakulla Co., FL	30.235	-84.302	10	7, 9-10, 20	FL
**Lake Iamonia (IAM *x *FL)**	17	Lake Iamonia at CR 155, Leon Co., FL	30.627	-84.291	10	1	FL
**Shepherd Spring (SHE *x *FL)**	18	Shepherd Spring at St. Marks National Wildlife Refuge, Wakulla Co., FL	30.123	-84.281	10	9-10, 27	FL
**McBride Slough (MCB *x *FL)**	19	McBride Slough at Bloxham Cutoff Rd., Wakulla Co., FL	30.239	-84.270	12	9-10, 43-44	FL
**Lake Overstreet (LOV *x *FL)**	20	Lake Overstreet at Alfred B. Maclay Gardens, Leon Co., FL	30.528	-84.257	10	1, 23	FL
**Newport Sulphur Spring (NEW *x *FL)**	21	Newport Sulphur Spring, Wakulla Co., FL	30.207	-84.179	10	2, 9, 15, 18	FL
**Natural Bridge (NAT *x *FL)**	22	Natural Bridge at Natural Bridge Rd., Wakulla Co., FL	30.284	-84.152	9	2, 18-19	FL
**Gambo Bayou (GAM *x *FL)**	23	Gambo Bayou at Lighthouse Rd. west of Stony Bayou Pool, Wakulla Co., FL	30.122	-84.148	9	9, 15	FL
**Tram Road (TRA *x *FL)**	24	Tram Rd., 2 km east of Plum Orchard, Wakulla Co., FL	30.140	-84.121	9	9, 15-16	FL
**Wacissa River (WAC *x *FL)**	25	Wacissa R., just north of Aucilla Wildlife Management Area, Jefferson Co., FL	30.341	-83.992	10	5-8	FL
**Buggs Creek borrow pit (BUG *x *FL)**	26	Buggs Creek barrow pit, FL	30.477	-83.844	2	35-36	FL
**Wolf Creek (WOL *x *FL)**	27	Wolf Creek at US 90, Jefferson Co., FL	30.532	-83.812	1	39	FL
**Mckey Park (MCK *x *GA)**	28	Mckey Park, Loundes Co., GA	30.864	-83.290	2	45	FL
**Bevil Creek (BEV *x *GA)**	29	Bevil Creek at Browns pond Loch Laurel Rd., GA	30.720	-83.244	2	3, 38	FL
**Robinson Creek (ROB *x *FL)**	30	Robinson Cr. at CR 131, Columbia Co., FL	30.321	-82.662	3	22	FL
**Ichetucknee blue hole (ICH *x *FL)**	31	Blue hole, Ichetucknee State Park, Columbia Co., FL	29.980	-82.758	2	32-33	FL
**Hillsborough River (HIR *x *FL)**	32	Hillsborough R. at Tampa, Hillsborough Co., FL	27.957	-82.465	11	22, 47	FL
**River Styx (RIS *x *FL)**	33	River Styx at CR 346, Alachua Co., FL	29.517	-82.222	2	‒	FL
**Saint Johns River (SJR2 *x *)**	34	Unnamed trib. of the St. Johns R., FL	‒	‒	3	22	FL
**Newnan's Lake (NEWn *x *FL)**	35	Newnan's Lake at southeast 16th Ave. boat ramp, Alachua Co., FL	29.637	-82.200	1	3	FL
**Everglades (EVE *x *FL)**	36	Everglades drainage, Dade Co., FL	25.552	-80.564	1	30	FL
**Bahama Swamp (BAH *x *SC)**	37	Bahama Swamp off Kato Bay Rd., Jasper Co., SC	32.356	-81.044	2	34	ACP
**Edisto River (CRO *x *SC)**	38	Edisto River, SC	33.085	-80.603	2	4, 46	ACP
**Back River (BAC *x *SC)**	39	Back R. at SSR 503, east of Goose Creek, edge of US Navy station, SC	32.967	-79.938	2	22	ACP
**Cooper River trib. (WAD1 *x *SC)**	40	Trib. to Cooper River at Wadboo, Berkeley Co., SC	33.201	-79.927	1	‒	ACP
**Waccamaw River (WAR1 *x *SC)**	41	Waccamaw River, SC	33.474	-79.188	1	‒	ACP
**Trib. to Waccamaw River (WAR2 *x *SC)**	42	Unnamed trib. to Waccamaw River, SC	33.506	-79.181	1	4	ACP
**Lumber River (LUM *x *SC)**	43	Lumber River, SC	33.661	-79.153	1	4	ACP
**Cane Branch (CAN *x *SC)**	44	Cane Branch on farm road at Francis Marion National Forest, Berkeley Co., SC	33.185	-79.527	1	21	ACP

Site names (numbers, localities, geographical coordinates), sample sizes (N), regional groups (Figure 1), and cyt*b* haplotypes are indicated. *Abbreviations*: *AL* Alabama, *Ave* avenue, *Co* County, *Cr* Creek, *CR* County Road, *FL* Florida, *GA* Georgia, *Hwy* highway, *MS* Mississippi, *Par* Parish, *R* River, *Rd* Road, *SC* South Carolina, *Trib* tributary.

†*x*-variables in codes range 1-12, representing each individual sequenced for this study.

We isolated DNA using the Qiagen DNeasy96 tissue protocol (Qiagen Sciences, Maryland, USA). We amplified the mitochondrial cytochrome *b* (cyt*b*) gene for each sample by PCR using Hrbek et al.’s [50] L14725 - H15982 primer pair. We also sequenced nuclear ribosomal protein S7 (*RPS7*) introns 1 and 2 and exon 2 for subsamples of 1-3 *H. formosa* individuals/site for up to 4 random sites per region within each clade (based on our multilocus gene tree; see Results), and Poeciliidae sp. samples. We amplified *RPS7* using a nested PCR design, with primers 1 F - 3R.24 in the first reaction followed by internal primers 1 F.2 - 2R.67 and 2 F.2 - 3R in subsequent reactions [51]. Primers 1 F and 3R are from Chow and Takeyama [52]. Amplification conditions, sequencing reactions, clean-up, and sequence visualization followed Unmack et al. [51]. We aligned sequences manually while viewing electropherograms in Sequencher™ 4.10 (Gene Codes Corp.). All sequences generated in this study were deposited in GenBank (accession numbers: KF632895-KF633114, cyt*b*, and KF633115-KF633133, *RPS7*). Alignment lengths were 1140 nucleotide base pairs (bp) for cyt*b* and 876 bp for *RPS7*. We supplemented these data with orthologous ingroup sequences from Lake Pontchartrain, Louisiana (N = 1) and Everglades, Florida (N = 1) retrieved from GenBank (Additional file 1: Table S1). Thus, our sampling encompassed *H. formosa* from 44 georeferenced sites (Table 1). In total, the final cyt*b* database we analyzed consisted of 223 *H. formosa* and 3 Poeciliidae sp. sequences that we collapsed into unique haplotypes in DnaSP 5.10 [53]. To create a multilocus alignment, we collated individuals with cyt*b* and *RPS7* sequences and joined them with analogous sequences from potential outgroups. Except multilocus and haplotype-based tests, analyses used the full cyt*b* database (sometimes divided among groups); iteratively adding/removing samples with missing data (usually <5 bp) had no qualitative effect on results.

**Figure 2:**
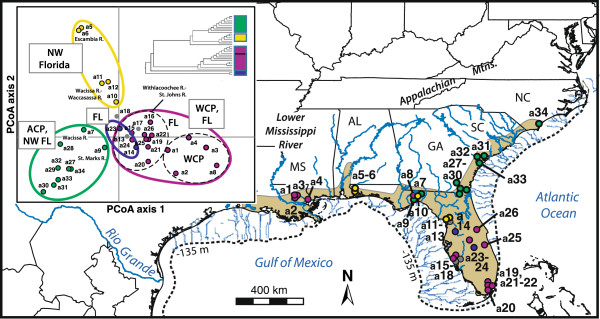
**Nuclear allozyme phylogeography.** Map of clades inferred from neighbor-joining analysis of unbiased Nei’s *D* from 11 allozyme loci in [42]. Inset graphic: corresponding tree topology with assigned clade colors, as well as the results of principal coordinates analysis (PCoA) of allele frequency data for the populations, which confirm the neighbor-joining relationships.

We also obtained data for 11 nuclear protein-encoding allozyme loci described in [42] (excluding locus *Idh-2* due to missing data for several populations), representing 948 *H. formosa* from 34 sites across the range (a1-a34, Figure 2; N = 12-36 individuals/site) that we georeferenced in decimal degrees. Putative loci included *Aat, Adk*, *Gpi-2*, *G3pdh*, *Idh-1*, *Ldh-2*, *Mdh-2*, *Mpi*, *Pgm-1*, *Pgm-2* and *6-Pgd*. Baer [42,54] showed these loci to be at Hardy-Weinberg equilibrium, so we included all 11 loci in analyses.

### Ecological niche modeling

To test the prediction that *H. formosa* experienced a southward LGM range contraction and a northward range expansion from LGM to present, consistent with the expansion-contraction model, we conducted ecological niche modeling using the maximum entropy (Maxent) approach implemented in MAXENT 3.3.3 k [55]. The Maxent method predicts locations of suitable habitat based on environmental-climatic characteristics and identifies abiotic and biotic factors that may limit species present-day distributions. We used Maxent because it exhibits high predictive performance [56].

We based our GIS-based niche-modeling analyses on 259 georeferenced *H. formosa* sites (Additional file 1: Figure S2) collated from our field collections (Table 1) and Global Biodiversity Information Facility (http://www.gbif.org) records. Sampling covered the species known distribution and was beyond sufficient given ≥10 sites permit accurate niche model construction [57]. From WorldClim (http://www.worldclim.org; [58]), we assembled GIS coverages with a resolution of 30 arc-seconds (1 km^2^) for 19 bioclimatic environmental predictor variables (Additional file 1: Table S2) for present-day climates (1950-2000), plus analogous paleoclimatic data layers reconstructing past LGM (21 ka) and LIG (140-120 ka) environments. The LGM dataset (resampled from 2.5 arc-minutes resolution) was derived from the CCSM3 global circulation model of the Paleoclimate Modelling Intercomparison Project Phase II (PMIP2; [59]). The LIG dataset (originally 30-s resolution) was derived from climate simulations in Otto-Bliesner et al. [25]. Layers were clipped to an extent of 24.5°N-35°N and 95°W-106°W prior to analyses. Unfortunately, high-resolution data on freshwater environments is not available for all three time-periods modeled, although such data would aid niche-based modeling of freshwater organisms. However, the CCSM3 data estimate environments over continental shelf areas exposed during the LGM; thus, we were able to reproject our full models over subaerial LGM landmass within the modeled area, which was critical for testing the expansion-contraction model. We ran niche models using different combinations of predictor variables to broadly explore the utility of different variables and their effects on model prediction. Ultimately, we included all 19 data layers in the final modeling procedures, because models showed no evidence of over-fitting and MAXENT is robust to correlations among variables (Additional file 1: Supplement S1).

We predicted the current (0 ka) geographical distribution of *H. formosa*, by generating an ecological niche model in MAXENT using the program’s default settings (e.g. 10^4^ background points, logistic model with habitat suitability between 0 and 1), except we increased the number of iterations to 5000 to ensure convergence and we averaged results over 10 replicate crossvalidation runs. All data were used for model testing and training. We reprojected the resulting niche models on LGM and LIG layers and interpreted areas with high-predicted LGM bioclimatic suitability as most-likely refugial areas. Under the expansion-contraction model, we expected areas of predicted occurrence to be reduced during the LGM relative to LIG and present-day, restricted to a southern refugium. By contrast, refugia located on either side of the Apalachicola River would support vicariance-northeast colonization, and a pattern of range stability through time would support southern crossroads. Model evaluation was based on threshold-independent measures of performance, area under the Receiver Operating Characteristic curve (AUC) statistics; scores closer to 1 (maximum) indicate higher predictive ability and AUC > 0.5 indicates better-than-random model prediction [56]. We generated response curves showing the impact of each variable alone on MAXENT prediction, and we studied 'multivariate environmental similarity surfaces’ (MESS) output by the program to assess extrapolation and effects of novel environments on prediction.

### Population structure and genetic diversity

We evaluated DNA polymorphism levels by calculating segregating sites (*S*), *h*, *Hd*, and π in DnaSP for the full *H. formosa* cyt*b* dataset, as well as populations and SAMOVA groups (see Results) that met a threshold-sampling criterion of N ≥ 8. Due to many samples not meeting our N ≥ 8 threshold-sampling criterion, mtDNA data were not used to test predicted genetic diversity patterns. Watterson’s estimator (*θ*_W_; from *S*) of *θ* was calculated overall and for SAMOVA groups. Additionally, we calculated uncorrected pairwise mtDNA p-distances between individuals and groups in MEGA 5 [60].

We analyzed the mtDNA data to test for patterns of spatial-genetic subdivision predicted by the various phylogeographical hypotheses. The expansion-contraction model predicts genetic drift should have created distinct lineages subdivided between refugia—if multiple, allopatric refugia existed—especially over repeated glacial cycles [2,15]. However, if northward recolonization occurred from a single southern refuge via 'leading-edge colonization,’ this should be reflected in a genetic distinction between the two areas, separating established (expanded) from blocked interior populations/lineages [2,9]. If the recolonization wave passed through spatial constraints, e.g. coastlines, we expect to identify most recent common ancestors (MRCA) through the coalescent and genetic distinctions that mark the location of barriers encountered outside of a refuge [61]. For what we term the 'vicariance-northeast colonization’ hypothesis (uniting Coastal Plain vicariance e.g. [29,32] and northeast colonization [42] elements), we tested for genetic barriers in the Apalachicola River-region between ACP-FL and WCP samples, and between the Suwannee and Hillsborough Rivers along Florida’s Gulf Coast (our sampling analog to the area of Baer’s [42] Waccasassa-Withlacoochee break). However, we expected genetic barriers to be absent within the ACP [42]. By contrast, a southern crossroads scenario would be supported by a pattern of no phylogeographical structure [21].

We tested these predictions using Monmonier’s [62] algorithm, implemented in BARRIER 2.2 [63], to identify genetic 'barriers’ across a Delaunay triangulation network overlaying the sampling sites, based on maximum Tamura–Nei genetic distances calculated in Arlequin 3.5 [64] (1000 nonparametric permutations). To assess barrier support, we calculated bootstrap proportions from 100 bootstrapped barriers generated in BARRIER using 100 bootstrapped Tamura–Nei distance matrices made in PopTools [65]; bootstrap values >50 indicated strong support. Next, we used spatial analysis of molecular variance (SAMOVA), implemented in SAMOVA 1.0 [66], to define population clusters maximizing the proportion of total genetic variance due to differences among geographical groups (Φ_CT_). We performed SAMOVAs across *K =* 2-8 groups using pairwise differences, drawing from any of 100 initial random conditions. We independently tested the grouping schemes indicated by the SAMOVA and BARRIER analyses by conducting analyses of molecular variance (AMOVA) in Arlequin (1000 nonparametric permutations). Because BARRIER and SAMOVA are sensitive to small sample sizes, we pooled adjacent sites until an N ≥ 8 threshold-sampling criterion was reached per population, yielding a 22-population subset.

We also conducted additional spatial analyses. Using AMOVA, we tested for significant genetic partitioning among drainages. Here, we capitalized on our dense north Florida sampling and split populations across seven drainages, including the Ochlockonee (collections 7-15, 17, and 20), St. Marks (16, 19, and 21-22), Apalachee plus Goose Creek Bays (18, 23-24), Aucilla (25-27), Suwannee (28-31), Hillsborough (32), and St. Johns Rivers (34-35). We employed GENALEX 6.3 [67] to examine predictions about patterns of isolation-by-distance, using Mantel tests [68] for correspondence between mtDNA genetic distances [*F*_ST_/(1 - *F*_ST_)] and geographic distances [*ln* (km)] among sites at rangewide and FL-region levels (N ≥ 8 threshold-sampling; 10^4^ permutations; relationships confirmed using linear regression). We used straight-line geographic distances, not river distances, because several sites were bodies of water (e.g. lakes) that are isolated from any nearby rivers. Based on our niche models (see Results), we predicted much of the range would lack isolation-by-distance; however, under vicariance-northeast colonization, only ACP populations should lack isolation-by-distance [42].

We tested for genetic diversity patterning consistent with the expansion-contraction model using analyses of the allozyme dataset. Coalescent theory predicts that northward postglacial expansion from reduced *N*_e_ should have created genetic signatures of population expansion in recently founded (recolonized) populations. These include excesses of rare alleles and low-frequency mutations, reduced genetic diversity (homozygosity, shorter demographic histories) resulting in negative latitudinal clines, and a lack of isolation-by-distance (reviewed in [4,9,69,70]). Conversely, long-term stable refugia should display isolation-by-distance and higher genetic diversity. We tested for predicted latitudinal clines in diversity by calculating expected heterozygosity (*H*_e_; Nei’s expected heterozygosity) over all loci in GENALEX (from allele frequencies), then testing for a negative relationship with latitude [4,9,17] using linear regression models in PAST 2.02 [71]. An absence of clinal diversity patterning would be more consistent with a southern crossroads scenario. We tested whether allozyme *H*_e_ and private allelic richness (*h*_p_) were greater within the niche modeling-inferred refugial populations (see Results) than throughout the putatively recolonized modern range using Mann-Whitney and Kolmogorov-Smirnov tests in PAST (due to non-normality, non-homogeneous variance). Using similar nonparametric tests, we tested whether WCP or ACP diversity was significantly lower than that of other populations combined, or populations within the putative refuge. And we tested for expected patterns of (lack of) isolation-by-distance using Mantel tests of unbiased Nei’s *D* genetic distances and *ln* geographic distances between populations, calculated in GENALEX at rangewide, WCP, ACP, FL, and within-refuge levels.

### Historical demography

We tested for population expansions predicted by the expansion-contraction model (as per above) at levels of regional groups, SAMOVA groups (see Results) and local populations (N ≥ 8 threshold size) using complementary cyt*b* analyses. We considered the expansion-contraction model 'strongly’ supported where at least two statistical tests supported expansion. First, we estimated Fu’s *F*_S_, and *R*_2_, and their 95% confidence intervals using coalescent simulations in DnaSP (testing significance with 10^4^ replicates). To distinguish population expansion from purifying or positive selection, we tested each group, and the full cyt*b* database, for mtDNA neutrality using McDonald and Kreitman’s [72] test and coalescent simulations of Fay and Wu’s *H*[73] in DnaSP (10^4^ replicates). We used Poeciliidae sp. as the McDonald–Kreitman test outgroup; other potential outgroups from Additional file 1: Table S1 yielded identical results (unpublished data). Second, we conducted mismatch distribution tests for regions/groups with no apparent subdivision (WCP, ACP and SAMOVA-group levels) in Arlequin (1000 parametric bootstrapping iterations) to see if pairwise nucleotide difference frequencies rejected sudden- or spatial-expansion models (given similar results, only sudden-expansion results are presented). We calculated Harpending’s raggedness index (*r*[70]) as a test statistic measuring goodness-of-fit to the mismatch distributions. We calculated the time in generations since population expansion (*t*) using estimated expansion parameters and the equation τ = 2 μ*t* (τ = coalescent time in mutations, μ = mutation rate/locus/year based on our Bayesian cyt*b* rate estimate; see Results); *t* was converted to time in years using generation time. We expected mean *t* and/or 95% confidence intervals to overlap the LGM.

### Phylogenetic relationships and coalescent-dating analyses

We expected multiple Pleistocene refugia to be supported by allopatric clades/unique networks [4,15] with strong nodal/parsimony support, whereas unique clades or intra-network structuring between refugia inferred from niche modeling and recolonized areas would support leading-edge effects [2,9]. Another phylogenetic prediction is that derived haplotypes (mutations at phylogeny/network tips) should become geographically localized outside of refugia with ancestral network populations found within refugia [61]. The placement of one or more network roots outside of the inferred refugia would be consistent with a model of allele surfing [61] or extinction of trailing expansion edges [9]. Here, southern crossroads is a null model with no obvious phylogeographical patterning within a single population-lineage.

We inferred relationships among *H. formosa* cyt*b* haplotypes with nodal support using maximum-likelihood tree searches and bootstrap searches (500 pseudoreplicates) in GARLI 0.97 [74], partitioning the data by codon position, ((1 + 2), 3). We analyzed our multilocus dataset using similar GARLI runs partitioning the data into cyt*b* codon-position subsets and an *RPS7*-gene subset, unlinking parameters across subsets. GARLI runs relied on DNA substitution models selected using the decision theory algorithm in DT-ModSel [75] (Supplement S1). We compared GARLI results to phylogenetic results from Bayesian coalescent-dating analyses (below) with similar site models. Because incomplete lineage sorting can obscure intraspecific phylogenetic relationships, we derived a 'species tree’ for our cyt*b* haplotypes using the 'Minimize Deep Coalescences’ method [76]. Although crude, this method increases probability of obtaining accurate population trees using even a single locus and yields insights into the degree of lineage sorting [77]. We also inferred phylogenetic relationships among populations from the allozyme dataset. We imported unbiased Nei’s *D* estimates (above) into PAUP* 4.0b10 [78] and used them to construct a midpoint-rooted neighbor-joining tree with total-distance branch lengths. We tested the genetic distinctiveness of the resulting clades in multivariate space using principal coordinates analysis (PCoA) conducted in GENALEX. Last, we inferred networks of cyt*b* and *RPS7* haplotype relationships with connections exceeding 95% parsimonious probability in TCS 1.21 [79].

We evaluated temporal diversification by estimating *H. formosa*-Poeciliidae sp. divergence, *H. formosa* population/clade divergence and other node ages using coalescent-dating analyses of our multilocus matrix in BEAST 1.74 [80]. To ensure convergence, we ran three independent searches (MCMC = 10^8^, sampled every 4000 generations; burn-in = 10^7^) using identical priors and relaxed, uncorrelated lognormal molecular clocks. We linked tree models but partitioned the data into cyt*b* codon-position subsets ((1 + 2), 3) and an *RPS7*-gene subset and unlinked clocks and parameters across subsets. Coalescent constant population size tree priors were used. We set uniform priors on rates drawn on by the lognormal relaxed clock that, for cyt*b*, spanned protein-coding mtDNA gene substitution rates reported for teleost fishes ('fish rate’ = 0.017-0.14 × 10^-8^ subs/site/yr, per-lineage; refs. in [81,82]), and for *RPS7* spanned an arbitrary range of reasonable rates (1.0 × 10^-10^-1.0 × 10^-8^ subs/site/yr) consistent with nDNA rates for freshwater fishes (unpublished data). By including *Poecilia* (subgenus *Limia*) outgroups in our analysis, we were able to add a calibration point constraining the split between *P. (L.) domicensis* (from Cuba) and *P. (L.) vittata* (Hispaniola) to 17-14 Ma based on *Poecilia* (*L.*) phylogeny [83] and geological dates for the separation of Cuba and Hispaniola [84], following Doadrio et al. [85]. We calibrated this node using a lognormal prior (mean in real space = 1, log standard deviation = 1.25, offset = 14). We used similar calibrations to model basal *Pseudoxiphophorus* divergence between 11-5 Ma, following Agorreta et al. [86], and to model the tree root age (Poeciliidae) to 39.9 Ma with an extended tail (log standard deviation = 2.0) based on the oldest fossils known for the family from the Paleocene-Eocene Maiz Gordo and Lumbrera formations, Argentina [87]. We summarized posterior parameter distributions and calculated parameter-trace effective sample sizes (ESS) in TRACER (http://tree.bio.ed.ac.uk/software/tracer). We archived our sequence alignments and maximum-likelihood and Bayesian tree results in TreeBASE (Submission 14718; http://purl.org/phylo/treebase/phylows/study/TB2:S14718).

### Hypothesis testing

We used coalescent simulations of mtDNA in Mesquite 2.73 [88] to statistically discriminate among phylogeographical scenarios representing the expansion-contraction model and two alternatives. This approach guards against the possibility that stochastic coalescences might make it more difficult to detect a signature of one or more models [43,44]. Population tree models in the simulations (described in Results, Supplement S1) spanned a total time (*t*_Total_) equal to tree depths of 3.741 × 10^6^ generations, derived by converting the mean intraspecific time to the most recent common ancestor (*t*_MRCA_) estimate from BEAST from absolute time to coalescent time (=1.247 Ma/*T*). We scaled branching intervals so that they summed to *t*_Total_. We estimated overall *N*_e_ for simulations (assumed equal to ancestral *N*_e_) by summing female *N*_e_ (*N*_ef_) estimates from *θ* values, calculated for each SAMOVA-inferred population using the equation *θ* = 2*N*_e_μ (for mtDNA, μ = cyt*b* rate estimated in BEAST). Thetas were derived from *θ*_W_ (above), and from Bayesian Θ estimates for SAMOVA groups, averaged from three replicate MIGRATE-N 3.1.3 [89] runs each using 1 long chain (3 × 10^8^) and adaptive heating (chains = 1, 1.5, 3, 10^4^; Supplement S1). We also estimated effective numbers of female migrants per generation (*N*_ef_*m*) from mtDNA in MIGRATE-N. Populations (internal branches) were modeled using groupings of subpopulations (areas, SAMOVA groups) appropriate to each hypothesis, and we scaled population-lineage sizes (internal branch widths) to their respective proportions of overall *N*_e_, so they summed to overall *N*_e_ at all time points [90]. Population-lineage sizes were equally distributed among tip branches (subpopulations) evolving from them. We conducted hypotheses testing in Mesquite by simulating 1000 mtDNA gene genealogies within the expansion-contraction model under neutral coalescence. For these simulated gene trees, we calculated the 'number of deep coalescences’ (nDC), a measure of gene tree-population tree discordance arising from incomplete lineage sorting [91]. We evaluated model fit by comparing nDC for the 'best’ cyt*b* maximum-likelihood gene tree to that from the simulated gene trees (treated as rooted). We rejected the expansion-contraction model in favor of an alternative by a one-tailed significance test (α = 0.05 level) [43].

## Results

### Ecological niche modeling

**Figure 3:**
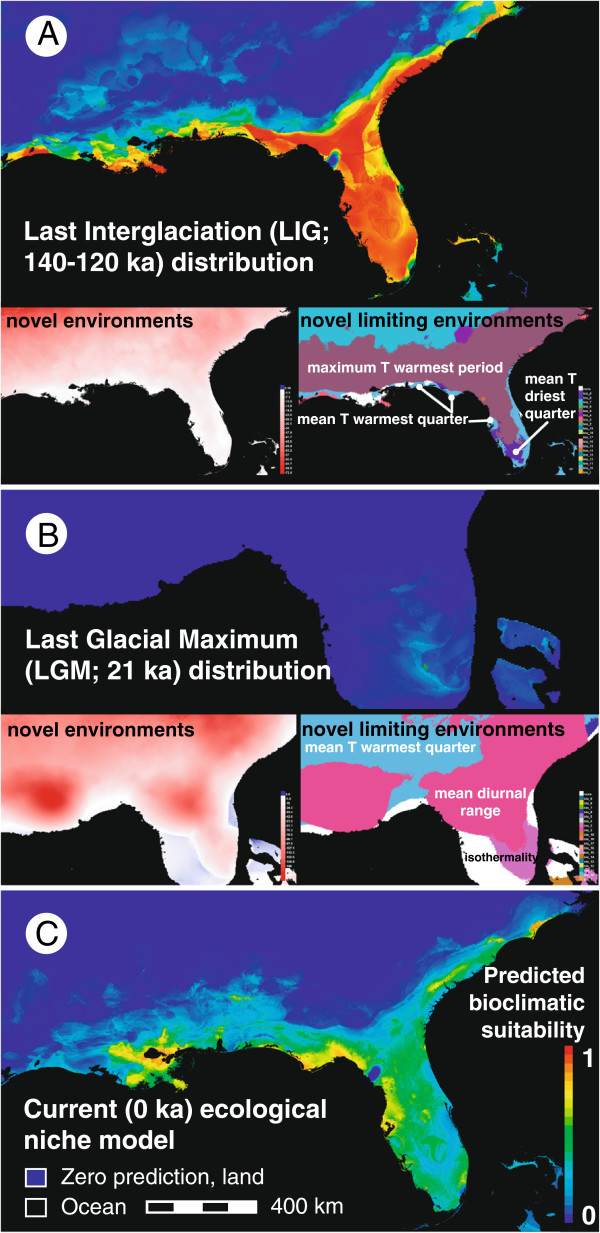
**MAXENT reconstructions of Pleistocene Last Interglaciation (LIG) and Last Glacial Maximum (LGM), and present-day distributions. A)** Reprojection of *H. formosa* ecological niche model **(C)** on paleoclimatic data layers representing LIG environments. **B)** Reprojection of *H. formosa* ecological niche model on colder/drier environmental conditions of the LGM, showing southward range contraction, possibly to 'microrefugia’. **C)** Present-day ecological niche model. Colors represent logistic ecological niche model scores and range from 0 (dark blue, indicating unsuitable subaerial areas) to 1 (100% bioclimatic suitability, thus higher predicted probability of species occurrence).

Ecological niche models predicting the species LIG, LGM, and present-day distributions are shown in Figure 3 and support expansion-contraction, not vicariance-northeast colonization or southern crossroads expectations. As exected, the paleoclimatic models predicted a wide LIG distribution (Figure 3A), drastic reduction in LGM bioclimatic suitability and southward range contraction to a refuge (Figure 3B), then northward recolonization of the modern range (Figure 3C). The putative refugium spanned roughly ≥1500 km^2^ area in an area of inland southwest Florida. The refugial area extended from the peninsula’s tip near Biscayne Bay (~25.15°N) to its southwestern slope west of the Immokalee Rise (50 m ASL), north to Charlotte Harbor (~28°N) and east across the Caloosahatchee Valley into south-central Florida (Figure 3B). The predicted bioclimatic suitability exceeded 0.079 (minimum logistic threshold). Additionally, we found that ACP and WCP-region suitability was similar across LIG/present-day interglacial models, as was a localized but distinct break in bioclimatic suitability recovered between the Suwannee and Withlacoochee Rivers.

Highly predicted (>80%) present and LIG ranges overlapped in patches around Lake Pontchartrain, Florida’s Apalachicola Bay-Apalachee Bay and western peninsula (Hillsborough River), and the northwest Atlantic Coast range margin. Under more conservative suitability criteria (logistic thresholds), present-day and LIG-modeled ranges covered >90% of the modern range. There were virtually no false negatives, but we obtained minor false positives where *H. formosa* does not occur (e.g. above the Fall Line, in the Bahamas).

Statistical model-evaluation analyses and comparisons of variable contributions supported these conclusions. Based on AUC scores from 10 replicates, models performed well with high discriminatory ability (LGM minimum training presence logistic threshold = 0.079, AUC = 0.920 ± 0.016; LIG minimum training presence logistic threshold = 0.022, AUC = 0.915 ± 0.018). Average test model gain (>1.5) also indicated >4-fold better-than-random prediction. The principal environmental predictor of occurrence was precipitation during warmest quarter, followed by temperature seasonality (Additional file 1: Tables S3-S4). MESS diagrams showed near-coastal regions experienced more novel climates during the LGM than the LIG, relative to present day, causing model extrapolation (Figure 3A,B). Still, novel environments were not a major issue: predictor variables most responsible for environmental novelty (novel limiting environments), e.g. isothermality, contributed ≤2.1% to MAXENT model prediction.

### Population structure and genetic diversity

We found substantial mean intraspecific mtDNA haplotype diversity (± standard deviation: *Hd* = 0.934 ± 0.006) but low mean nucleotide diversity (π = 0.0066 ± 0.009) and surprisingly shallow genetic divergence (mean cyt*b* p-distance = 0.66%, range: 0-1.61%), all of which are consistent with a recent, rapid population expansion for the species. Using DnaSP, we calculated an empirical estimate of *θ*_W_ = 0.0087 for all *H. formosa* populations combined. SAMOVA-group estimates of population mutation rate parameter were moderate, with *θ*_W_ ranging 0.0031-0.0053 (Additional file 1: Table S5)*.*

All three methods of spatial genetic analyses recovered three locations of genetic distinctions that defined four groups (*K*) within the species range (Figure 1). The groupings were strongly supported by bootstrap analysis (BARRIER BP = 55-99); significant spatially explicit among-group variance partitioning (SAMOVA P < 0.0001); and AMOVAs (Supplement S1). While SAMOVA and BARRIER analyses were not completely concordant, both algorithms recovered genetic barriers outside of the ecological niche modeling-inferred LGM refugium, and both inferred complex structuring between the Apalachicola delta and Aucilla River. These results are most consistent with leading-edge colonization or allele surfing (or other factors) during post-glacial range expansion. However, they do not firmly reject vicariance-northeast colonization: barriers were identified between WCP and ACP regions, but not FL and ACP; groups were divided north-to-south between the Suwannee and Hillsborough Rivers; and no ACP genetic barriers were inferred.

Most genetic barriers reflected breaks between drainages, and we also found significant among-drainage genetic partitioning in Florida (AMOVA: Among-group variation = 33.73%, Φ_CT_ = 0.34, P < 0.0001). The most striking SAMOVA-inferred subdivision was isolation of this northern Florida area from all other populations (WCP, south Florida peninsula, ACP). However, within northern Florida, SAMOVA connected geographically disjunct lakes/ponds (collections 13-15, 17, and 20), Womack Creek (10), and Robinson Creek (30), and linked disjunct Newnan’s Lake (35) and Tate’s Hell sites (7-8). BARRIER recovered contiguous groups (Figure 1) and split the WCP from other areas (barrier *ii*); however, given disparities with our other DNA and allozyme results, and barrier *ii*’s placement in a sampling gap, we interpreted the WCP group as a manifestation of the limitations of our mtDNA sampling.

Mantel tests indicated that these conclusions were not unduly influenced by spatial autocorrelation in cyt*b* variation , whether rangewide (*r* = -0.007, P = 0.492; regression R^2^ = 5 × 10^-5^, *t* = -0.11, P = 0.913) or only within Florida (*r* = -0.054, P = 0.391; regression R^2^ = 0.003, *t* = -0.74, P = 0.460). MtDNA isolation-by-distance was thus overall lacking, reflecting low diversity and alleles shared among regions/populations; for example, haplotype 22 was common to all three regions and most ACP populations shared haplotype 4 (Table 1 and S6). We noted a similar lack of nDNA isolation-by-distance, with *RPS7* haplotype 2 present in each study region (Additional file 1: Table S7). These results agree with expansion-contraction predictions as well as the independently derived niche models.

**Figure 4:**
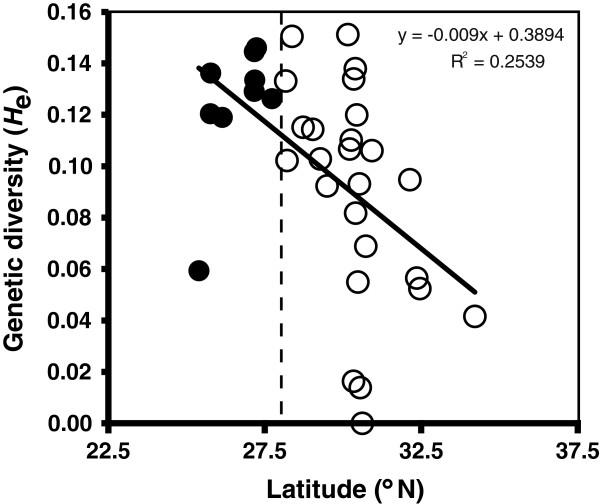
**Latitudinal cline in *Heterandria formosa *allozyme genetic diversity.** This linear regression model shows a strong negative relationship between allozymic genetic diversity (*H*_e_) and latitude (P = 0.002). Black dots indicate genetic diversity of sites below 28°N latitude (dashed line; the temperate-tropical transition), with bioclimatic prediction above the logistic threshold in our Last Glacial Maximum model (Figure 3B), i.e. within the putative LGM refugium.

Consistent with expansion from the niche-modeling inferred refugium, allozyme genetic diversity (*H*_e_) decreased with increases in latitude moving northward from the likely refugium (R^2^ = 0.254,*t* = -3.30, P = 0.002; Figure 4). Consistent with the inferred refugial location (Figure 3B), allozyme diversity was above average and statistically higher within, rather than outside, the putative refuge (WCP, ACP, 'outside’ populations combined), as judged by *H*_e_ (Table 2). However, *h*_p_ was not statistically different within-vs.-outside the putative refuge (Table 2). Consistent with the mtDNA results, Mantel tests of the 11 allozyme loci revealed significant isolation-by-distance in the putative refuge (*r* = 0.360, P = 0,014; regression R^2^ = 0.498, *t* = 2.815, P = 0.022). We also found that isolation-by-distance was significant rangewide (*r =* 0.236, P = 0.001; regression R^2^ = 0.056, *t* = 5.77, P < 0.0001) and within regions (see Supplement S1 for details) except the ACP (*r =* 0.031, P = 0.323; regression R^2^ = 0.001, *t* = 0.18, P = 0.855). Allozyme polymorphism levels also supported the vicariance-northeastern colonization prediction of relatively lower ACP diversity (Table 2).

**Table 2:** Nonparametric tests of spatial-diversity predictions based on allozyme variation

				**Mann-Whitney tests**		**Kolmogorov-Smirnov tests**		
**Comparison (group 1-vs.-group 2)**	**Mean 1**	**Mean 2**	**N**	***U***	***z***	**P**	***D***	**P**
***H***_**e**_								
**Within-vs.-outside putative refuge**	0.122	0.089	34	59	-2.287	0.022*	0.55	0.016*
**ACP-vs.-rest of range**	0.071	0.108	34	36	-2.740	0.006**	0.69	0.003**
**ACP-vs.-putative refuge**	0.071	0.122	14	5	-3.065	0.002**	0.80	0.002**
**WCP-vs.-rest of range**	0.072	0.105	34	49	-1.559	0.119 ns	0.43	0.244 ns
**WCP-vs.-putative refuge**	0.072	0.122	16	8	-2.332	0.019*	0.70	0.0251*
***h***_**p**_								
**Within-vs.-outside putative refuge**	0.036	0.015	34	99	-1.168	0.243 ns	0.41	0.141 ns
**ACP-vs.-putative refuge**	0.000	0.036	14				0.30	0.738 ns
**WCP-vs. putative refuge**	0.000	0.036	16				0.40	0.472 ns

*H*_e_, expected heterozygosity; *h*_p_, private allelic richness; N, number of populations compared; *P < 0.05; **P < 0.01; ns, not significant.

### Historical demography

**Figure 5:**
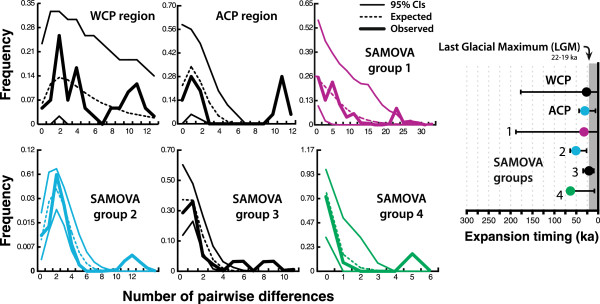
**The six graphs at left show the frequency distributions of the numbers of pairwise cytb differences among H. formosa individuals for each SAMOVA-inferred population group and regional group, with confidence intervals on expected values derived from parametric bootstrapping (10**^4 ^**iterations) in Arlequin.** Mean estimated timing of population expansions (circles) and 95% confidence intervals from parametric bootstrapping (bars) are plotted at right, with grey shading indicating time since onset of the LGM.

Historical-demographic analyses of cyt*b* variation strongly supported predicted patterns of population expansions among regions and population groups. The *R*_2_ statistic was significant (P < 0.0001) and positive for the species, and for each regional group, SAMOVA group, and population analyzed (Table 3, S5). One positive Fu’s *F*_S_ estimate was significant (P < 0.02), indicating a genetic bottleneck in the WCP region (Table 3). Further testing rejected the possibility that *R*_2_ and *F*_S_ deviations from migration-drift equilibrium resulted from positive or purifying selection (full cyt*b* database, McDonald–Kreitman test, P = 0.33; mean *H* = -0.035, 95% confidence intervals: [-14.93, 5.45], P = 0.33; see Additional file 1: Table S5 for other results). Mismatch distributions [70,92] were mostly unimodal or smoothly descending with modal numbers of differences between haplotypes ranging 0-2 for WCP, ACP, and SAMOVA groups (except small peaks of divergent haplotypes at low frequencies; Figure 5). Moreover, based on Harpending’s *r*, we failed to reject models of sudden demographic expansion in all cases (Table 3), indicating the coalescent model accurately accounted for observed polymorphism patterns. From τ, we inferred a Late Pleistocene timing of intraspecific expansions (20.4-61.6 ka; Table 3), with the onset of expansion preceding the LGM by 0-40 ka, and overlapping estimates suggesting concurrent expansions since ~33-10 ka. Except in SAMOVA group 2, we failed to reject LGM expansions because confidence interval included values less than 22 ka (Table 3; Figure 5).

**Table 3:** **Neutrality and mismatch distribution tests for population expansions within *Heterandria formosa***

**Group**	**N**	***F***_**S**_	***R***_**2**_	***r***	**τ**	**Expansion time (ka)**
**WCP region**	8	0.288**	0.201**	**0.240 ns**	1.195 [0.000, 10.393]	24.5 [0.0, 213.4]
**ACP region**	9	0.241	0.178**	**0.169 ns**	1.498 [0.000, 3.119]	30.8 [0.0, 64.0]
**SAMOVA group1**	10	0.218	0.169**	**0.061 ns**	1.672 [0.000, 9.023]	34.3 [0.0, 185.3]
**SAMOVA group 2**	115	-0.267	0.090**	**0.058 ns**	2.408 [1.256, 3.031]	49.4 [25.8, 62.2]
**SAMOVA group 3**	51	-0.053	0.108**	**0.095 ns**	0.992 [0.492, 1.615]	20.4 [10.1, 33.2]
**SAMOVA group 4**	23	0.081	0.133**	**0.482 ns**	3.000 [0.410, 3.172]	61.6 [8.4, 65.1]

Sample sizes and coalescent simulation results for Fu’s *F*_S_, *R*_2_, Harpending’s raggedness index (*r*), and mutation time parameter τ calculated from a sudden-expansion demographic model. Multiple tests supported population expansions for each group of populations (boldface); one test supported a population bottleneck (underlined). Population expansion dates (thousands of years ago, ka) were estimated from τ using a per-locus mutation rate (μ = 8.12 **×** 10^**-**6^) converted from the BEAST cyt*b* estimate. Regional results are only presented for regions with no SAMOVA-inferred subdivision. Brackets contain 95% confidence intervals; ns, not significant; **P < 0.0001 (P < 0.02 for Fu’s *F*_S_).

### Phylogenetic relationships and coalescent-dating analyses

**Figure 6:**
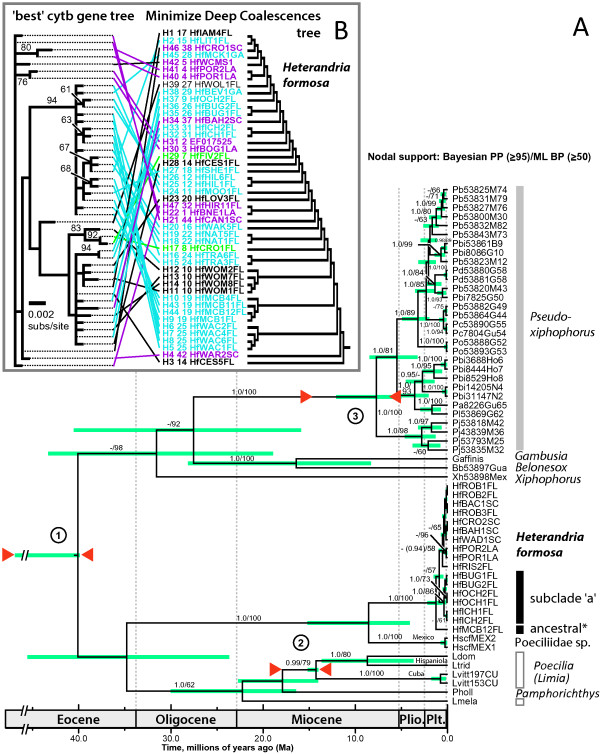
**Chronogram of *Heterandria formosa *and related poeciliid species diversification, and gene tree results. A)** Chronogram resulting from Bayesian relaxed-clock coalescent-dating analysis in BEAST based on mitochondrial cyt*b* and *RPS7* variation. Tip labels are sequence codes including population, site number, and specimen code for each individual sequenced (details in Figure 1 and Table 1). Node bars (dark blue) are 95% highest posterior densities for node ages. The analysis included three lognormally modeled fossil/biogeographic calibration points (red triangles enclose bounds of constraints). Mean node ages of interest are discussed in detail in the text. Nodal support values are of the form: Bayesian posterior probabilities (PP; ≥95)/maximum-likelihood bootstrap proportions (BP; ≥50). **B)** Comparison between the 'best’ gene tree of *H. formosa* cyt*b* haplotypes and the 'Minimize Deep Coalescences’ species tree (at right) inferred from the haplotype tree using Maddison and Knowles’ [76] method, with BP ≥ 50 by each node.

Within our 223-cyt*b* dataset, we found 47 unique *H. formosa* haplotypes distinguished by 59 segregating sites, and two unique Poeciliidae sp. haplotypes. Subsampling across regions yielded a final multilocus alignment of cyt*b* and *RPS7* sequences for 17 *H. formosa* and 2 Poeciliidae sp., plus outgroups in Additional file 1: Table S1. Maximum-likelihood (Additional file 1: Figure S3) and Bayesian phylogenetic searches (Figure 6A; below) on the multilocus alignment recovered identical topologies with a monophyletic *H. formosa* comprised of a single lineage lacking strongly supported (BP ≥ 70 and PP ≥ 95) phylogenetic structure except a subclade (subclade 'a’) representing the Ochlockonee River in part, Aucilla River, and the Suwannee River in part; otherwise, geographical structure was insignificant.

Several features of the sequence data supported predictions derived from the expansion-contraction model and were not consistent with the predictions of a vicariance-northeast colonization model. For one, no deep intraspecific east-west breaks were supported. This is not because there were no deep phylogenetic breaks detected; the predicted sister relationship between Poeciliidae sp. and *H. formosa* (maximum cyt*b* p-distance = 9.01%) was strongly supported (Figure 6A, Additional file 1: Figure S3), indicating a deep split between Mexico and the Atlantic Plain. Our 'best’ cyt*b* haplotype gene tree (*ln L* = -2000.8824) revealed shallow variation and a close Ochlockonee-Aucilla-Suwannee relationship, and supported differentiation (albeit incomplete) of SAMOVA groups 2 and 3. This topology differed greatly from the 'Minimize Deep Coalescences’ topology, indicating substantial incomplete lineage sorting (Figure 6B).

**Figure 7:**
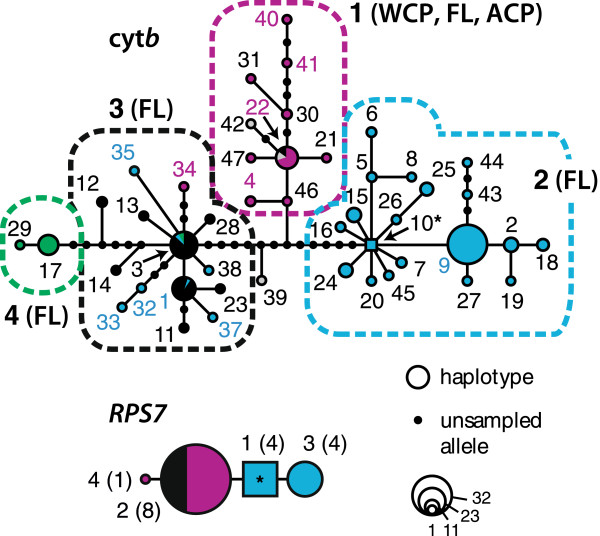
**Statistical parsimony network relationships among cyt*b *and *RPS7 *haplotypes (numbered).** Networks were defined in TCS based on a 95% parsimony criterion. Network circles indicate haplotypes scaled according to their frequency (smallest colored circles = 1 sample; scale applies to cyt*b* network; *RPS7* haplotype frequencies are given in parentheses), lines represent 1 mutation step between haplotypes, and dotted lines enclose distinct network regions (numbered, with regions in parentheses). Colors represent haplotype identities, by SAMOVA-inferred population groups (*K* = 4) shown in Figure 1.

Consistent with shallow variation and a single population-lineage experiencing recent expansion-contraction dynamics, cyt*b* and nuclear *RPS7* haplotypes formed single networks (Figure 7) and *RPS7* sequences from the Apalachicola River-Apalachee Bay region were separated by only 1-3 mutations from haplotypes spread throughout the rest of the species range. Consistent with expansion-contraction, populations (alleles) in the putative LGM refuge (haplotypes 22, 30, and 47) were mostly recovered as ancestral/basal in the inferred cyt*b* gene tree (100%) and network (66.7%). By contrast, outside of the putative LGM refugium (44 haplotypes) we found mostly derived populations (tip alleles) in the gene tree (81.8%) and network (70.5%). The cyt*b* network reflected the population structure inferred from the SAMOVA analysis and had two cases of star-like patterns with branches radiating out from inferred root-haplotype 10 (McBride Slough) and haplotype 3 (Cessna Pond, collection 14). Also, many (39%) network alleles were unsampled, indicating alleles potentially lost to glacial-stage extinctions.

Allozyme phylogenetic analysis recovered four shallow, spatially overlapping clades that were also genetically distinct during PCoA multivariate projection, with axes 1 and 2 explaining 39.9% and 28.8% of the genetic variance, respectively (Figure 2). Consistent with expansion-contraction predictions, allozyme results corroborated SAMOVA-inferred population structure, with a north-south break between the Suwannee and Hillsborough Rivers, separating a group of northwestern plus south Florida populations from all others. Allozyme results also agreed with other phylogenetic results: the northwest Florida allozyme clade matched the strongly supported subclade 'a’ in Figure 6A. Contrasting our DNA sequence-based results, and consistent with vicariance-northeast colonization, neighbor-joining and PCoA recovered ACP populations as a genetically and geographically distinct clade/group. However, against vicariance-northeast colonization expectations, intraspecific clades/groups were surprisingly not split east-to-west near the Apalachicola River-region (Figure 2) or St. Mary’s River, as suggested by [42].

The model resulting from multilocus coalescent-dating in BEAST (Figure 6A; *ln L* ± standard error = -10,054.902 ± 0.118) yielded a geometric mean *t*_MRCA_ of *H. formosa* samples dated to 1.247 Ma [0.620, 2.178] in the Early-Middle Pleistocene. The *t*_MRCA_ estimate for subclade 'a’ was 0.377 Ma [0.153, 0.713]. Bayesian posterior distributions for these *t*_MRCA_ estimates strongly rejected LGM divergence (P < 0.05; <5% of posterior samples estimated a *t*_MRCA_ for this node younger than 22 ka). Thus, the deepest population structuring (interpopulation divergence) within *H. formosa* appears to have initiated *before* the LGM. We estimated divergence between Poeciliidae sp. and *H. formosa* at 8.478 Ma [3.939, 14.913] in the Late Miocene-Early Pliocene; confidence intervals for this split were much wider than the *t*_MRCA_s above but we still statistically rejected Pleistocene divergence (P < 0.05). Parameter-trace ESS scores >600 indicated thorough Markov chain mixing. Our BEAST runs estimated a geometric mean evolutionary cyt*b* rate of 7.12 × 10^-9^ substitutions/site/yr, and an *RPS7* rate of 1.01 × 10^-9^ substitutions/site/yr.

### Hypothesis testing

By summing regional group *N*_ef_ estimates based on empirical mtDNA *θ*_W_ (Additional file 1: Table S1) and Θ (range: 0.00106-0.00360) estimates, we respectively inferred overall *N*_ef_ ≈ 6.05 × 10^5^ and 3.53 × 10^6^. We also inferred migration rates among SAMOVA groups (mean *N*_ef_*m* range: 1.184-2.887); however, Bayesian posterior *N*_ef_*m* distributions peaked at the lower limit of resolution, with 95% confidence intervals including zero. Setting ancestral *N*_e_ equal to the *N*_ef_ estimates above, we ran separate simulations testing three hypotheses modeled as hypothetical population trees (see Additional file 1: Supplement S1): (1) a null, 'fragmented ancestor’ model representing the expansion-contraction model, with a single size-constant ancestral population evolving through the Pleistocene before experiencing a 90% LGM reduction in *N*_e_ (range contraction), and subsequent colonization and diversification of local subpopulations (15-0 ka; summing to current *N*_e_) while simultaneously expanding from a source population (south Florida refugium located by ecological niche modeling; Figure 3B); (2) a 'vicariance-northeast colonization’ model with a Early Pleistocene ancestor split basally east-west of Apalachicola River/Mobile Bay and ACP populations arising spontaneously post-LGM (15-0 ka) from a Florida refuge (colonized from a Saint Johns River source); and (3) a 'four-refugia’ vicariance model with SAMOVA population groups (Figure 1, results below) diverging from a Pleistocene ancestor. The number of deep coalescences for our 'best’ cyt*b* gene tree fit into the population trees for each hypothesis was 9 for the null model, 14 for vicariance-northeast colonization, and 27 for four-refugia. Results of both simulation sets failed to reject the expansion-contraction hypothesis in favor of alternative models (P < 0.001), thus our gene tree is more consistent with a scenario of LGM population contraction and postglacial re-expansion than either of the alternative vicariance models.

## Discussion

Our results failed to reject most expansion-contraction model predictions (66.7% of tests; summarized in Additional file 1: Table S8). Indeed, several independent lines of evidence from geospatial and genetic data give concordant results consistent with this model. Here, we explore these results.

Ecological niche modeling suggests that *H. formosa* underwent range contraction to a single LGM refugium in the southwest Florida peninsula, an area that underwent less environmental-climatic change than the northern parts of the species range (Figure 3B). Paleoclimatic data, climate models and pollen records indicate southwest Florida maintained ~0-4°C lower temperatures, wetter tropical climates (+400-800 mm/yr annual precipitation) and mixed southern pine/hardwood forest to open woodland vegetation during the LGM [11,27,93], relative to today. By contrast, LGM conditions in northern Florida and the 'mainland’ Gulf-Atlantic plains were colder/drier with extreme ~8-10°C drops in winter temperatures, 400 mm/yr lower annual precipitation, and patchy northern forest and grasslands vegetation [27,93,94]. Combined with these data, our results strongly suggest *H. formosa* were extirpated from their northern range and survived the LGM in bioclimatically suitable peninsular areas, areas that, in fact, were the only parts of eastern North America that did not experience significantly colder winter and summer LGM temperatures [94].

While one paleobotanical synthesis suggested this putative refugial area was nothing more than arid sand-dune scrub at the time [13], other data [11] indicate that at this time south Florida was semiarid, containing suitable freshwater stream and wetland/marsh habitats where cold-intolerant freshwater organisms persisted. This deduction is consistent with pollen data showing aquatic macrophytes such as *Brasenia*, *Isoëtes*, *Myriophyllum*, *Typha* and *Sparganium* occurring within the Florida peninsula during the LGM [95].

Comparing the predicted LGM refuge of *H. formosa* with its much wider modern distribution (Figures 1 and 3C), we suggest that *H. formosa* rapidly recolonized most or all of its preceding range to the north (e.g. of the LIG, Figure 3A) during postglacial climatic 'amelioration.’ After 16-15 ka, eastern North American rivers underwent transitions from braided to meandering channel types, during which wetter-than-modern early Holocene conditions may have increased wetlands and river discharge [96]. Conditions from this time onward may thus have favored expansion into parts of the species range, even though temperature and precipitation did not stabilize near their modern levels until somewhat later, ~9-6 ka [93]. Such northward post-glacial expansions were unlikely to have been slowed by Younger Dryas cooling 12-11 ka (i.e. potentially negative effects on aquatic habitats), which did not affect areas below ~38°N latitude ([96], refs. therein). As a corollary, we reject vicariance-northeast colonization-predicted range dynamics and our results also seem to run counter to rangewide population stasis and connectivity expected under the southern crossroads hypothesis [21]; apparently, smaller areas of the North American Coastal Plain (south Florida) may have experienced prolonged ecological stability than in Mediterranean Basin coastal plains.

Ecological niche modeling assumes that relationships between present distributions, abiotic factors, and ecological interactions remain more or less at equilibrium, that species physiological limits have changed less than the magnitude of changes in abiotic variables, and that biotic features of a species’ niche affect historical distribution less than large-scale changes in abiotic conditions [18,22,23]. We cannot evaluate all of these assumptions, nor can we exclude possible bias due to coastline effects i.e. major influence of the Bermuda high-pressure zone on coastal precipitation, which was among the variables of highest (>30%) predictive importance (Additional file 1: Tables S3-S4). Nonetheless, our niche-based modeling results provide meaningful insights into *H. formosa* biogeography. Our paleoclimatic models yield highly accurate predictions, e.g. AUCs >0.90 and our results are consistent with existing ecophysiological data. For example, modern *H. formosa* populations reach critical thermal minima at ~10°C [97] and live close to this cold-season limit in northern parts of their range. Coinciding with this value, 88% of the *H. formosa* localities (Additional file 1: Figure S2) had mean temperature of coldest quarter values ≥10°C (±0.1°C), and this variable had sharply higher effects on MAXENT prediction above 10°C (JCB, unpublished data). Given cold-season temperatures across the species entire northern range declined to -8 to 4°C during the LGM [27,93], it is reasonable to infer that glacial-stage extirpation occurred passively in these areas, with drought and colder winter temperatures reducing survival. In addition, the assumption of ecological niche conservatism on these spatial and temporal scales seems justified for this species. Differences in thermal tolerances among *H. formosa* populations from different parts of their range are minimal (~2 °C; [97]) compared to the temperature differences induced by climatic oscillations of the last glacial cycle above; thus differential physiological adaptation to temperature regimes likely has not had a confounding effect on our inferences. Our analyses of DNA sequence data and re-analyses of nuclear allozyme loci reinforce the paleoclimatic data to support expansion-contraction dynamics. In particular, the negative latitudinal cline suggests a glacial refugium in south Florida and northward recolonization [4,9,15]. Admixture in south Florida due to secondary contact of diverged lineages, i.e. following 'vicariance’ expected under vicariance-northeast colonization, or recent dispersal mediated by episodic connections (e.g. flooding, axial-valley connections) can be ruled out for two reasons. First, there is a distinct lack of phylogenetic divergence and, second, seven allozyme alleles in south Florida are private alleles not shared with populations in other areas. These data are also inconsistent with southern crossroads dynamics supported elsewhere, in which no clinal diversity patterns were witnessed [21]. That our phylogeographical results also support expansion-contraction dynamics agreeing with the niche models also suggests that the assumption of ecological niche conservatism through time is essentially correct for *H. formosa*.

Of course, it is difficult to discount the possibility of 'microrefugia’ [98] in small habitat pockets elsewhere. This is because our niche-based models predict responses to prevailing macroclimatic conditions, not microclimatic conditions below the data-layer grain (1 km^2^) or within local habitats. If any secondary refugium existed, the most likely candidate area would seem to have been the Tallahassee area, where we also found one private allozyme allele.

Several of our results agree with previous studies [42,54]. Similar to Baer [42] and despite extreme distances of >2000 km between our collection localities and those in the allozyme dataset, we find *H. formosa* are characterized by shallow genetic divergences and a single population-lineage (e.g. mean mtDNA p-distance = 0.66%; Figures 6 and 7). Combined with high *Hd* but low π estimates, this suggests recent postglacial expansion with too limited time for recovery to allow accumulation of large sequence divergence [29], and/or high historical gene flow levels. Also congruent with [42], we infer no genetic barriers within the Atlantic Coastal Plain. This is consistent with Baer’s 'northeast colonization’ hypothesis (ACP populations colonized following an Early Pleistocene high-sea stand following 1.5 Ma), which he argued was also supported by lower allozyme diversity and a lack of ACP isolation-by-distance [42] (see below). *Heterandria formosa* also contains four significantly differentiated mtDNA population groups subdivided outside of the putative refugium, or between the refugium and recolonized areas (Figures 1, 2 and 7). The latter break, inferred between the Suwannee and Hillsborough Rivers (i.e. SAMOVA groups 1 vs. 2-4; Figures 1 and 7), corresponds roughly to a north-to-south break between Gulf-draining Waccasassa and Withlacoochee Rivers described by Baer [42]. This break creates a clade of Louisiana plus south Florida samples in our phylogenetic and multivariate ordination analyses of the allozyme-frequency data, and Baer’s (Figure 2; though our results are based on different genetic distances). This Suwannee-Hillsborough break thus appears robust to different data types and methods and is shallow in both studies (e.g. ~0.6-0.8% mtDNA divergence) implying recently limited gene flow between these rivers. Perhaps more importantly, the Suwannee-Hillsborough break falls (with other genetic barriers) outside the predicted LGM refugium but is not correlated with deep phylogenetic divergence. This is consistent with the action of density-dependent processes, such as priority effects e.g. [2,9], or allele surfing [61], operating during leading-edge recolonization. Given their patchy distribution [39,42] and propensity for explosive population growth [99], it is reasonable to assume that *H. formosa* quickly fill new habitats once they arrive and that when local patches become extinct they are recolonized from nearby populations. Thus if northward postglacial expansion occurred through small numbers of colonists, alleles might have surfed along a relatively quickly expanding wave front. Our niche-based models suggest postglacial genetic divergence between these rivers may have partly been maintained by lack of predicted suitable habitat between these rivers (Figure 3).

By contrast, we find several points of nuclear-mitochondrial discordances, indicating differences between studies. For example, allozyme and mtDNA results yield mixed support for the expansion-contraction prediction that isolation-by-distance should occur within the refugium, but not outside of it, due to a longer demographic timeline and geographically restricted dispersal within the refuge. Allozymic variation exhibits nearly rangewide isolation-by-distance in our study and Baer’s [42]. Baer [42] also inferred populations were at or near drift-migration equilibrium, using a 2-dimensional stepping stone model with cross-peninsula gene flow. Global *N*_e_*m* estimates were high (>4, range: 2.5-37.5), suggesting local *H. formosa* populations have not evolved independently but that gene flow has importantly shaped their evolution [42]; and in a second study, *N*_e_*m* within and across two peninsular Florida rivers ranged >3-400 [54]. In Baer [42], ACP populations were exceptional, forming a relatively homogeneous monophyletic group with no isolation-by-distance consistent with recent colonization, consistent with our mtDNA and allozyme findings. However, contrasting these patterns, we find: a lack of mtDNA isolation-by-distance across the range; *N*_e_*m* suggesting zero on-going mtDNA gene flow (see Supplement S1); and ACP populations as indistinct from other regions (i.e. nonmonophyletic; Figures 2, 6 and 7). Incongruent with Baer’s [42] allozyme results, the historical signal of the mtDNA genome also does not indicate east-to-west genetic differentiation between WCP and ACP regions (Figure 1).

Such nuclear-mitochondrial discordances are traditionally explained by several factors. The first and most common explanation is balancing selection (e.g. during genetic hitchhiking) mainly on allozymes [100], some of which (particularly metabolic protein loci) are known to evolve non-neutrally. However, this is rejected outright by the data: Lewontin-Krakauer tests on the allozymes [42,54] and multiple tests of mtDNA neutrality (e.g. Additional file 1: Table S5) reject the hypothesis of selection operating in either genome, as does the concordant lack of deep phylogenetic structure within either marker-class. A second explanation is that these discordances arise from the contrasting modes of transmission, evolution, and resolution of these marker-types; this could plausibly explain a lack of isolation-by-distance in the mtDNA, but isolation-by-distance in the allozymes. Compared with haploid, maternally transmitted mtDNA, allozymes are diploid and evolve roughly 10× slower with ~4-fold larger *N*_e_ and coalescence times (independent of mutation rate), and higher gene-flow rates [29,101,102]. As a consequence, mtDNA provide a higher-resolution view of population structure, e.g. at finer spatial scales (supported by our data), and more likely reflect homogenizing effects of gene flow hence lack of isolation-by-distance. By contrast, due to lower resolution, allozymes may display isolation-by-distance in northern areas even when expansion occurred from a single southern refugium, if postglacial expansion(s) did not involve leptokurtic (long-distance) dispersal e.g. reviewed in [2,4,9]. Regarding discordant migration patterns, allozyme *N*_e_*m* estimates and *N*_e_ estimates seem preferable in the case of *H. formosa* (mtDNA typically drastically underestimate *N*_e_ in high-gene flow species [29]). Still, their large coalescence times mean allozyme-inferred *N*_e_*m* may reflect averages across transmission routes over four times the history of mtDNA, rather than current processes. Thus, we interpret our results and Baer’s [42] as indicating that historical gene flow (high *N*_e_*m*) among populations nearing migration-drift equilibrium, and large *N*_e_, has influenced *H. formosa* phylogeographical structuring. This could partly explain the low observed genetic divergences, but can we reconcile this scenario with the isolation-by-distance findings, taken at face value? Taking such a scenario as compatible with the expansion-contraction model assumes most population structure has arisen since the LGM and that sufficient time has passed for *H. formosa* allozyme loci to reach equilibrium. This is supported by the data if we assume *Nm* = 10 (similar to [42], or higher [54]) and that populations must have achieved migration-drift equilibrium since the LGM, or *G* = 57,000 generations (19 ka*T). Given our *N*_ef_ (~6 × 10^5^-3 × 10^6^) and ~1.2 Ma intraspecific *t*_MRCA_ estimates, the number of generations required to approach equilibrium is recent, ~60,829 generations, and since the LGM based on the equation *G* = 1/(2 *m* + 1/(2*N*_e_)) [103]. Thus, the above interpretation seems reasonable in light of population genetics theory; equilibrium could have arisen even faster in this species, as postglacial recolonization and population expansion can rapidly generate population structuring [61].

Another factor potentially explaining our results is sex-biased migration. *Heterandria formosa* exhibit strongly female-biased sex ratios due to higher male mortality rates, as demonstrated by predation trials [104] and otolith ring counts (JT, personal observation). In light of this, female dispersal/gene flow should outpace male dispersal even assuming equal dispersal propensities and rates between sexes. Thus, aside from differences in marker resolution, female-biased dispersal has likely also contributed importantly to nuclear-mitochondrial discordance above, specifically lack of mtDNA isolation-by-distance. This is because, under female-based dispersal, females carry both mtDNA and male/female nuclear genes out of populations while males are effectively philopatric (geographically restricted), thus we expect matrilineal alleles to more likely homogenize among localities while nuclear genomes remain site-dependent [29]; this would result in a relatively weaker matrilineal signal of isolation-by-distance. Unfortunately, we cannot reliably empirically evaluate effects of female-biased survival and dispersal on our other genetic results given comparable metrics influenced by geographical distance (e.g. linearized *F*_ST_) are not estimable from the mtDNA and allozyme-frequency data, and low *RPS7*-gene sampling restricts possible mtDNA-nDNA comparisons. Last, consistent with the observation that deep coalescences appear to influence the *H. formosa* mtDNA/gene tree (e.g. Figure 6B), patterns of discordance between markers herein may to some extent reflect historical processes of incomplete lineage sorting in addition to the homogenizing effects of gene flow [significant when *Nm* > 4 (reviewed in [29]), as in *H. formosa*]. In future studies, sampling multiple, unlinked nuclear loci will be necessary to tease apart the relative contributions of these two opposing forces within *H. formosa* using coalescent-based models.

In addition to the geospatial and genetic analyses above, our phylogenetic and historical-demographic results also met predictions of the expansion-contraction model. While our inferences regarding ancestral derived/populations were limited by reduced south Florida sampling, phylogenetic and spatial patterns of ancestral/derived populations (alleles) between the phylogeny and network analyses congruently follow expectations derived from the model (e.g. Figure 4, Additional file 1: Table S8), and star-like patterns in the network suggest a history of population expansions. Our argument that *H. formosa* fits the expansion-contraction model is also reinforced by mismatch analyses and mtDNA genetic equilibrium tests (Fu’s *F*_S_, *R*_2_), which fail to reject demographic expansion models (e.g. for SAMOVA-inferred population groups; see text; Table 3, S5; Figure 5) and thus capture genetic signatures of predicted historical changes in *N*_e_ containing information about the record and timing of expansion [61,70,92]. The 95% confidence intervals on expansion parameter τ suggest the onset of expansions has overlapped the LGM in most population groups and regions analyzed, and lower 95% confidence intervals extending into the present may be indications that expansion is on-going or ceased only recently, e.g. in north Florida (Table 3; Figure 5). Because balancing selection seems not to have been at play (as per above), low-frequency peaks in our mismatch distributions likely reflect secondary Holocene expansions rather than selection (Figure 5).

These demographic results generally agree with genetic patterns in European taxa (despite possibly earlier onset of *H. formosa* expansions) that are inferred to have experienced postglacial population expansions e.g. [4,17]; however, in our case, integrating niche-based modeling and genetic analyses provides an additional check that it is reasonable to conclude *H. formosa* expansion has been both spatial and demographic (Figure 3; Table 3). Interestingly, our mtDNA genetic equilibrium tests only point to recent population bottlenecking within WCP samples (Table 3) spanning extensive wetlands and swamps of the Mississippi River Delta, a major outlet of postglacial meltwater flow and alluvial deposition. This could reflect a small population surviving the LGM in one or more WCP microrefugia not identified in our paleoclimatic models; however, no evidence exists that a population-lineage experienced drift in isolation in the WCP since the LGM (e.g. Figure 6B). We thus interpret this bottleneck as a result of founder events during postglacial recolonization.

Although the distribution of *H. formosa* spans nearly the entire lowland Coastal Plain (Figure 1), its genetic patterns do not completely coincide with those described in several other species with overlapping present-day distributions. At least 20 species, including 50-67% of freshwater fishes and turtles examined to date, display pronounced east-west Apalachicola phylogeographical [29-32] and distributional [33] breaks. In four freshwater fishes—bowfin (*Amia calva*) and three sunfishes (*Lepomis gulosus*, *Lepomis microlophus*, and *Lepomis punctatus*)—this pattern has been linked to vicariant isolation in upper reaches of different drainage basins that were reduced in size by eustatic high-stands 50-80 m ASL of the Pliocene-Early Pleistocene interglacials [105,106]. Thus, major phylogenetic divergence(s) should be expected in this region in as-yet unsampled freshwater taxa. Remarkably, however, neither patterns of multilocus phylogenetic structuring or mtDNA-population structuring, nor a coalescent simulation perspective on neutral mtDNA evolution, support such a pattern within *H. formosa*. Variation at protein loci in *H. formosa* also departs from this vicariance model, revealing shallowly diverged and geographically overlapping population groups/lineages without deep genetic breaks at predicted barriers. While the common pattern of Gulf drainages containing Atlantic-coast haplotypes (but not vice versa) is present in the allozymes (Figure 2), neither mtDNA nor allozyme data support clear east-west Apalachicola splits.

Our estimated *t*_MRCA_ suggests intraspecific diversification of *H. formosa* has coincided with Pleistocene diversification within *A. calva*, which is codistributed with *H. formosa* in the Coastal Plain but ranges much more widely across eastern North America (including the Mississippi and St. Lawrence River basins). This indicates that recent isolation within constraints of Pleistocene-Holocene (rather than earlier) drainage geomorphology, e.g. drainage divides, has influenced genetic variation of both *H. formosa* (i.e. genetic distinctiveness of mtDNA at AMOVAs structured by Florida drainages) and *A. calva*. However, additional studies will be necessary to test whether these species diversification has actually been synchronous, and to evaluate finer-scale patterns relative to drainage basins. It would also be interesting to examine whether Coastal Plain freshwater fish species that are presently codistributed with *H. formosa* responded to Late Pleistocene disruptions in climate and sea-level with similar range-shifting responses, possibly involving refugia; however, the only species in which effects of Pleistocene expansion-contraction dynamics have been rigorously tested is *H. formosa* (this study). Here, the eastern/Atlantic intraspecific lineages (Florida peninsula-Atlantic seaboard) present in many freshwater taxa will likely present good candidates for testing for range expansion-contraction dynamics similar to *H. formosa*. For example, the eastern/Atlantic lineages recovered by Bermingham and Avise [32] exhibit <2-4% genetic divergences, indicating Pleistocene *t*_MRCA_s (assuming the standard 2%/Myr clock for vertebrate mtDNA [101]), and phylogroups or private alleles unique to the Florida peninsula. While *ad hoc* explanations that interspecific differences in the position of the Apalachicola break (particularly geographical distributions of the eastern lineages) resulted from unique histories of dispersal and range-shifting in response to Pleistocene climatic fluctuations have been advanced [29,31,32], the actual pattern of such dynamics remains an unanswered question. Additional studies similar to ours of previously studied fish taxa, e.g. in [32], as well as understudied aquatic plants, insects, freshwater fishes (e.g. *Fundulus seminolis*, *Jordanella floridae*, etc.), and crayfishes from the Florida peninsula will therefore permit testing the generality of the scenario of historical range-shift and population dynamics uncovered within *H. formosa*, as well as general predictions of the expansion contraction model [15], within the Gulf-Atlantic Coastal Plain freshwater biota.

## Conclusions

Understanding historical responses of species to the climatic and sea-level fluctuations of the Late Pleistocene has emerged as a major goal of ecology, evolution and biogeography [2,4,15,29]. We find the evolutionary history of *Heterandria formosa* indicates southward LGM range contraction and large-scale postglacial expansion, as predicted under the Pleistocene expansion-contraction model [15]. Species-specific expansion-contraction dynamics may therefore have played a more general role in shaping the evolutionary history of Coastal Plain biota than previously thought. By adding to considerable variation surrounding common biogeographical themes recovered among even codistributed species in this region, our results bolster growing appreciation for the complexity of phylogeographical patterning in North America’s southern refugia. Our study demonstrates the benefits of taking an integrative approach to biogeographical hypotheses generation and testing (e.g. more realistic hypotheses testing through niche modeling and statistical phylogeography) and showcases how similar approaches can be used by biologists in the future to gain further insights into the evolutionary history of the Gulf-Atlantic Coastal Plain biota, as well as the important role of this region as a dynamic refuge during the last glacial cycle. Indeed, a better understanding of the tempo and mode of species responses to the Pleistocene glacial-interglacial cycles will likely aid predicting and managing ecosystem responses to present-day and future climate change [6].

### Availability of supporting data

Sequences are deposited in GenBank (accession numbers: KF632895-KF633114, cyt*b*, and KF633115-KF633133, *RPS7*). Sequence alignments and phylogenetic trees are available in the TreeBASE repository: http://purl.org/phylo/treebase/phylows/study/TB2:S14718.

## Competing interests

The authors declare that they have no competing interests.

## Authors’ contributions

MS and JCB conceived of the project. JCB directed project design and coordination, conducted molecular laboratory work, performed genetic and ecological niche modeling analyses, archived data in online repositories, and wrote the manuscript. MS conducted field and molecular laboratory work, discussed analyses, and revised the manuscript. MLLV and JT made field collections and revised the manuscript. JBJ discussed molecular laboratory work and helped revise and structure the manuscript. All authors have read and approved the final manuscript.

## Supplementary Material

Additional file 1 **Data supplement.** Contains supplementary **Tables (S1-S9)** and **Figures (S1-S3)** and an additional description of methods and results (Supplement S1). Click here for file
