# Supplementary material for: Paleoclimatic modeling and phylogeography of least killifish, Heterandria formosa: insights into Pleistocene expansion-contraction dynamics and evolutionary history of North American Coastal Plain freshwater biota
Source: BMC Evol Biol. 2013 Oct 9;13:223. doi: 10.1186/1471-2148-13-223 (PMC3851817; doi:10.1186/1471-2148-13-223)
Supplement: Additional file 1 — Data supplement. Contains supplementary Tables (S1-S9) and Figures (S1-S3) and an additional description of methods and results (Supplement S1). [file 1471-2148-13-223-S1.docx]

**Additional file 1: Data supplement**

**for Bagley JC, Sandel M, Travis J, Lozano-Vilano ML, Johnson JB:** Paleoclimatic modeling and phylogeography of least killifish, *Heterandria formosa*: insights into Pleistocene expansion-contraction dynamics and evolutionary history of North American Coastal Plain freshwater biota. *BMC Evolutionary Biology* 2013*.*

**Table S1 Collections data including species name, specimen codes and IDs, GenBank accession numbers, and corresponding references for previously published mitochondrial and nuclear DNA sequences used to supplement data gathered in this study**

|  |  |  |  |  | **GenBank numbers** | |
| --- | --- | --- | --- | --- | --- | --- |
| **Number** | **Species** | **Specimen code^†^** | **ID used in this study** | **Locality, country** | cyt*b* | *RPS7* |
| **1** | ***Heterandria formosa*** | EF017525 | EF017525 | Lake Pontchartrain, Louisiana (site 2, Table 2), USA | EF017525 [1] | ‒ |
| **2** | ***Heterandria formosa*** | AF412125 | AF412125 | Everglades, Florida (site 36, Table 2), USA | AF412125 [2] | ‒ |
| **3** | ***Belonesox belizanus*** | MNCN/ADN 53897 | Bb53987Gua | Guatemala | JQ612908 [3] | JQ613101 [3] |
| **4-5** | ***Gambusia affinis*** | 33528 (cyt*b*), LLSTC 04578 (*RPS7*) | Gaffinis | ‒ | NC_004388 [4] | HM443941 [5] |
| **6** | ***Limia dominicensis*** | Ldom | Ldom | ‒ | EF017533 [1] | ‒ |
| **7** | ***Limia melanogaster*** | Lmela | Lmela | ‒ | EF017534 [1] | ‒ |
| **8** | ***Limia tridens*** | Ltrid | Ltrid | ‒ | EF017535 [1] | ‒ |
| **9** | ***Limia vittata*** | 153CU | Lvitt153CU | La Boca Lagoon, Camaguey, Cuba | FJ178765 [6] | ‒ |
| **10** | ***Limia vittata*** | 197CU | Lvitt197CU | Abra River, Juventud Island, Cuba | FJ178766 [6] | ‒ |
| **11** | ***Pamphorichthys hollandi*** | Pholl | Pholl | ‒ | EF017538 [1] | ‒ |
| **12** | ***Pseudoxiphophorus "bimaculatus"*** | MNCN/ADN 31147 | Pbi31147N2 | Nicaragua | JQ612784 [3] | JQ613056 [3] |
| **13** | ***Pseudoxiphophorus "bimaculatus"*** | STRI 14205 | Pbi14205N4 | Nicaragua | JQ612790 [3 | JQ613058 [3] |
| **14** | ***Pseudoxiphophorus "bimaculatus"*** | STRI 3688 | Pbi3688Ho6 | Honduras | JQ612791 [3] | JQ613059 [3] |
| **15** | ***Pseudoxiphophorus "bimaculatus"*** | STRI 8444 | Pbi8444Ho7 | Honduras | JQ612792 [3] | JQ613060 [3] |
| **16** | ***Pseudoxiphophorus "bimaculatus"*** | STRI 8529 | Pbi8529Ho8 | Honduras | JQ612793 [3] | JQ613061 [3] |
| **17** | ***Pseudoxiphophorus "bimaculatus"*** | MNCN/ADN 53861 | Pbi53861B9 | Belize | JQ612863 [3] | JQ613082 [3] |
| **18** | ***Pseudoxiphophorus "bimaculatus"*** | STRI 8086 | Pbi8086G10 | Guatemala | JQ612904 [3] | JQ613097 [3] |
| **19** | ***Pseudoxiphophorus "bimaculatus"*** | MNCN/ADN 53823 | Pb53823M12 | Mexico | JQ612825 [3] | JQ613068 [3] |
| **20** | ***Pseudoxiphophorus "bimaculatus"*** | MNCN/ADN 53800 | Pb53800M30 | Mexico | JQ612803 [3] | JQ613064 [3] |
| **21** | ***Pseudoxiphophorus "bimaculatus"*** | MNCN/ADN 53820 | Pb53820M43 | Mexico | JQ612822 [3] | JQ613067 [3] |
| **22** | ***Pseudoxiphophorus "bimaculatus"*** | MNCN/ADN 53864 | Pb53864G44 | Guatemala | JQ612866 [3] | JQ613083 [3] |
| **23** | ***Pseudoxiphophorus "bimaculatus"*** | MNCN/ADN 53882 | Pb53882G49 | Guatemala | JQ612884 [3] | JQ613090 [3] |
| **24** | ***Pseudoxiphophorus "bimaculatus"*** | STRI 7825 | Pbi7825G50 | Guatemala | JQ612899 [3] | JQ613096 [3] |
| **25** | ***Pseudoxiphophorus "bimaculatus"*** | MNCN/ADN 53843 | Pb53843M73 | Mexico | JQ612845 [3] | JQ613076 [3] |
| **26** | ***Pseudoxiphophorus "bimaculatus"*** | MNCN/ADN 53825 | Pb53825M74 | Mexico | JQ612827 [3] | JQ613069 [3] |
| **27** | ***Pseudoxiphophorus "bimaculatus"*** | MNCN/ADN 53827 | Pb53827M76 | Mexico | JQ612829 [3] | JQ613070 [3] |
| **28** | ***Pseudoxiphophorus "bimaculatus"*** | MNCN/ADN 53831 | Pb53831M79 | Mexico | JQ612833 [3] | JQ613071 [3] |
| **29** | ***Pseudoxiphophorus anzuetoi*** | STRI 8226 | Pa8226Gu65 | Guatemala | JQ612906 [3] | JQ613099 [3] |
| **30** | ***Pseudoxiphophorus cataractae*** | STRI 7804 | Pc7804Gu54 | Guatemala | JQ612898 [3] | JQ613095 [3] |
| **31** | ***Pseudoxiphophorus cataractae*** | MNCN/ADN 53890 | Pc53890G55 | Guatemala | JQ612892 [3] | JQ613093 [3] |
| **32** | ***Pseudoxiphophorus cf. tuxtlaensis*** | MNCN/ADN 53832 | Pb53832M82 | Mexico | JQ612834 [3] | JQ613072 [3] |
| **33** | ***Pseudoxiphophorus diremptus*** | MNCN/ADN 53880 | Pd53880G58 | Guatemala | JQ612882 [3] | JQ613088 [3] |
| **34** | ***Pseudoxiphophorus diremptus*** | MNCN/ADN 53881 | Pd53881G58 | Guatemala | JQ612883 [3] | JQ613089 [3] |
| **35** | ***Pseudoxiphophorus jonesii*** | MNCN/ADN 53793 | Pj53793M25 | Mexico | JQ612796 [3] | JQ613062 [3] |
| **36** | ***Pseudoxiphophorus jonesii*** | MNCN/ADN 53835 | Pj53835M32 | Mexico | JQ612837 [3] | JQ613073 [3] |
| **37** | ***Pseudoxiphophorus jonesii*** | MNCN/ADN 53839 | Pj43839M36 | Mexico | JQ612841 [3] | JQ613074 [3] |
| **38** | ***Pseudoxiphophorus jonesii*** | MNCN/ADN 53818 | Pj53818M42 | Mexico | JQ612820 [3] | JQ613066 [3] |
| **39** | ***Pseudoxiphophorus litoperas*** | MNCN/ADN 53869 | Pl53869G62 | Guatemala | JQ612871 [3] | JQ613084 [3] |
| **40** | ***Pseudoxiphophorus obliquus*** | MNCN/ADN 53888 | Po53888G52 | Guatemala | JQ612890 [3] | JQ613092 [3] |
| **41** | ***Pseudoxiphophorus obliquus*** | MNCN/ADN 53893 | Po53893G53 | Guatemala | JQ612895 [3] | JQ613094 [3] |
| **42** | ***Xiphophorus helleri*** | MNCN/ADN 53898 | Xu5398Mex | Mexico | JQ612909 [3] | JQ613102 [3] |

Data on additional ingroup (*H. formosa*) sequences used for genetic analyses in this study are presented in the first two rows of this table. Note that additional site data for these sequences is provided in Table 1. The remaining rows present data on 72 additional gene sequences from 39 samples (tip taxa) from related Poeciliidae species, representing 23 additional ‘potential outgroup’ lineages that we incorporated into the DNA alignments used in our phylogenetic analyses in order to conduct an outgroup analysis (see below, and Methods section, for further details). Under ‘ID used in this study,’ we present phylogenetic tip names used to represent the sequences in our alignments and figures (also see TreeBASE Submission 14713).

^†^Specimen or sample number codes from reference study text or GenBank accession entry.

**Figure S1 paleo-bathymetric rivers data**

**
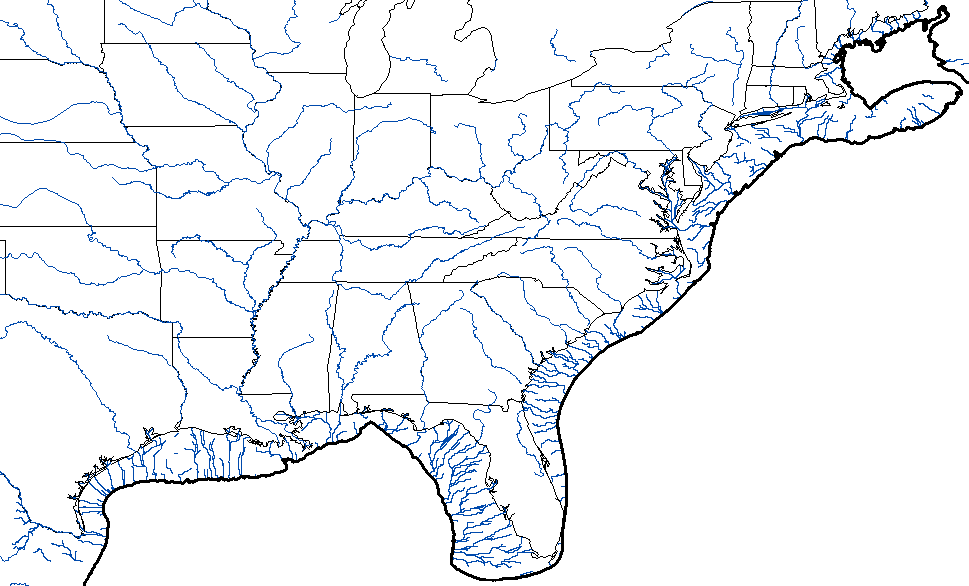
**

Credit: Peter J. Unmack (used with permission; see details in Supplement S1 below).

**Figure S2 Map of *Heterandria formosa* test data (occurrence data) used in ecological niche modeling analyses in MAXENT**

**
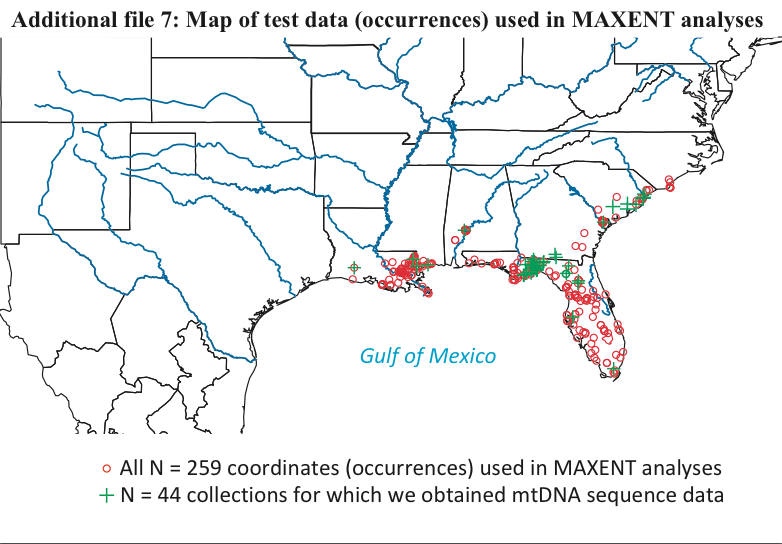
**

**Table S2 Environmental data variables used to construct ecological niche models in this study**

| **Variable #** | **Variable name** | **Description** |
| --- | --- | --- |
| **1** | **BIO1** | Annual mean temperature |
| **2** | **BIO2** | Mean diurnal range |
| **3** | **BIO3** | Isothermality |
| **4** | **BIO4** | Temperature seasonality |
| **5** | **BIO5** | Maximum temperature of warmest period |
| **6** | **BIO6** | Minimum temperature of coldest period |
| **7** | **BIO7** | Temperature annual range |
| **8** | **BIO8** | Mean temperature of wettest quarter |
| **9** | **BIO9** | Mean temperature of driest quarter |
| **10** | **BIO10** | Mean temperature of warmest quarter |
| **11** | **BIO11** | Mean temperature of coldest quarter |
| **12** | **BIO12** | Annual precipitation |
| **13** | **BIO13** | Precipitation of wettest period |
| **14** | **BIO14** | Precipitation of driest period |
| **15** | **BIO15** | Precipitation seasonality; standard deviation of averages of weekly precipitation |
| **16** | **BO16** | Precipitation of wettest quarter |
| **17** | **BIO17** | Precipitation of driest quarter |
| **18** | **BIO18** | Precipitation of warmest quarter |
| **19** | **BIO19** | Precipitation of coldest quarter |

This table describes the pool of all of the bioclimatic data (19 variables/layers, from [7] accessed through the WorldClim database) used as sources of data from which we drew during ecological niche model construction (details in main text and Supplement S1 below). What follow next in Tables S3-S4 are descriptions of the relative contributions of the environmental predictor variables to each MAXENT model.

**Table S3 Environmental variable importance to prediction for the model estimating the current ecological niche model and then reprojecting it on data layers representing environments of the Last Interglaciation (LIG; Figure 3A)**

| **Rank** | **Variable #/name** | **Percent contribution** | **Permutation importance** |
| --- | --- | --- | --- |
| **1** | BIO18 | 30.3 | 6.0 |
| **2** | BIO4 | 24.3 | 4.0 |
| **3** | BIO16 | 12.2 | 5.1 |
| **4** | BIO14 | 9.0 | 12.1 |
| **5** | BIO11 | 3.4 | 11.2 |
| **6** | BIO15 | 3.4 | 11.3 |
| **7** | BIO12 | 3.1 | 3.9 |
| **8** | BIO13 | 1.9 | 2.7 |
| **9** | BIO7 | 1.8 | 5.9 |
| **10** | BIO8 | 1.7 | 2.1 |
| **11** | BIO19 | 1.7 | 10.6 |
| **12** | BIO17 | 1.6 | 3.0 |
| **13** | BIO3 | 1.5 | 1.8 |
| **14** | BIO1 | 1.4 | 12.8 |
| **15** | BIO10 | 1.3 | 4.8 |
| **16** | BIO9 | 0.6 | 1.3 |
| **17** | BIO5 | 0.3 | 0.4 |
| **18** | BIO6 | 0.3 | 0.7 |
| **19** | BIO2 | 0.3 | 0.3 |

**Table S4 Environmental variable importance to prediction for the model estimating the current ecological niche model and then reprojecting it on data layers representing environments of the Last Glacial Maximum (LGM; Figure 3B)**

| **Rank** | **Variable #/name** | **Percent contribution** | **Permutation importance** |
| --- | --- | --- | --- |
| **1** | BIO18 | 36.0 | 16.6 |
| **2** | BIO16 | 10.4 | 6.1 |
| **3** | BIO11 | 9.6 | 20.4 |
| **4** | BIO4 | 8.2 | 5.7 |
| **5** | BIO7 | 8.0 | 6.1 |
| **6** | BIO14 | 6.5 | 10.4 |
| **7** | BIO15 | 3.5 | 6.8 |
| **8** | BIO8 | 2.5 | 2.9 |
| **9** | BIO12 | 2.3 | 2.0 |
| **10** | BIO10 | 2.3 | 6.4 |
| **11** | BIO13 | 2.2 | 2.1 |
| **12** | BIO3 | 1.8 | 1.1 |
| **13** | BIO19 | 1.5 | 7.7 |
| **14** | BIO17 | 1.5 | 0.7 |
| **15** | BIO5 | 1.3 | 0.6 |
| **16** | BIO9 | 0.8 | 0.9 |
| **17** | BIO6 | 0.6 | 0.2 |
| **18** | BIO1 | 0.5 | 2.7 |
| **19** | BIO2 | 0.4 | 0.5 |

**Table S5 MtDNA polymorphism levels and results of neutrality tests across lineage, regional group, SAMOVA group, and population levels**

| **Sampling** | | | **MtDNA polymorphism** | | | | | | **Neutrality tests** | | | |
| --- | --- | --- | --- | --- | --- | --- | --- | --- | --- | --- | --- | --- |
| **A)** | | | | | | | | | | | | |
| **Lineage** | **Nos.** | **N** | ***S*** | ***h*** | ***Hd*** | **s.d.** | **π**  **(×100)** | ***θ_w_*** | **MK test** | | ***H*** | |
| ***Heterandria formosa*** | ‒ | 220 | 59 | 44 | 0.934 | 0.006 | 0.660 | 0.00874 | 0.673 | | -0.042 | |
|  |  |  |  |  |  |  |  |  |  | |  | |
| **B)** | | | | | | | | | | | | |
| **Regional groups** | **Nos.** | **N** | ***S*** | ***h*** | ***Hd*** | **s.d.** | **π**  **(×100)** | ***θ_w_*** | **MK test** | | ***H*** | |
| **WCP** | 1-5 | 8 | 7 | 6 | 0.929 | 0.084 | 0.242 | 0.00237 | 0.675 | | -0.013 | |
| **FL** | 7-32, 34-36 | 203 | 50 | 36 | 0.926 | 0.007 | 0.634 | 0.00749 | 0.669 | | 0.054 | |
| **ACP** | 37-39, 42-44 | 9 | 12 | 4 | 0.821 | 0.101 | 0.461 | 0.00388 | 0.515 | | -0.005 | |
|  |  |  |  |  |  |  |  |  |  | |  | |
| **C)** | | | | | | | | | | | | |
| **SAMOVA groups** | **Nos.** | **N** | ***S*** | ***h*** | ***Hd*** | **s.d.** | **π**  **(×100)** | ***θ_w_*** | **MK test** | | ***H*** | |
| **group 1** | 1-4, 32, 36-39, 42, 44 | 10 | 15 | 6 | 0.889 | 0.075 | 0.480 | 0.00466 | 0.352 | | -0.069 | |
| **group 2** | 9, 11-12, 15-16, 18-19, 21-26, 28-29, 31 | 115 | 32 | 25 | 0.887 | 0.019 | 0.294 | 0.00530 | 0.341 | | -0.056 | |
| **group 3** | 10, 13-14, 17, 20, 30 | 51 | 19 | 9 | 0.697 | 0.042 | 0.195 | 0.00370 | 0.588 | | -0.xxx | |
| **group 4** | 7-8, 35 | 23 | 13 | 5 | 0.391 | 0.125 | 0.223 | 0.00309 | 0.758 | | -0.xxx | |
|  |  |  |  |  |  |  |  |  |  | |  | |
| **D)** | | | | | | | | | | | | |
| **Population (specimen no. code**†**)** | **No.** | **N** | ***S*** | ***h*** | ***Hd*** | **s.d.** | **π**  **(×100)** | ***θ_w_*** | ***F*_S_** | ***R*_2_** | **MK test** | ***H*** |
| **Five Points (FIV*x*FL)** | 7 | 9 | 1 | 2 | 0.222 | 0.166 | 0.019 | ‒ | 0.324 | **0.255**** | ‒ | ‒ |
| **Crooked River (CRO*x*FL)** | 8 | 10 | 0 | 1 | 0.000 | 0.000 | 0.000 | ‒ | ‡ | ‡ | ‒ | ‒ |
| **Womack Creek (WOM*x*FL)** | 10 | 9 | 10 | 6 | 0.889 | 0.091 | 0.336 | ‒ | 0.264 | **0.182**** | ‒ | ‒ |
| **Moore Lake (MOO*x*FL)** | 11 | 10 | 0 | 1 | 0.000 | 0.000 | 0.000 | ‒ | ‡ | ‡ | ‒ | ‒ |
| **Hill Swale (HIL*x*FL)** | 12 | 10 | 1 | 2 | 0.200 | 0.154 | 0.018 | ‒ | 0.332 | **0.244**** | ‒ | ‒ |
| **Trout Pond (TRO*x*FL)** | 13 | 10 | 0 | 1 | 0.000 | 0.000 | 0.000 | ‒ | ‡ | ‡ | ‒ | ‒ |
| **Cessna Pond (CES*x*FL)** | 14 | 9 | 1 | 2 | 0.222 | 0.166 | 0.019 | ‒ | 0.329 | **0.255**** | ‒ | ‒ |
| **Wakulla Springs (WAK*x*FL)** | 16 | 10 | 3 | 4 | 0.644 | 0.152 | 0.082 | ‒ | 0.475 | **0.213**** | ‒ | ‒ |
| **Lake Iamonia (IAM*x*FL)** | 17 | 10 | 0 | 1 | 0.000 | 0.000 | 0.000 | ‒ | ‡ | ‡ | ‒ | ‒ |
| **Shepherd Spring (SHE*x*FL)** | 18 | 10 | 2 | 3 | 0.511 | 0.164 | 0.049 | ‒ | 0.461 | **0.227**** | ‒ | ‒ |
| **McBride Slough (MCB*x*FL)** | 19 | 12 | 4 | 4 | 0.636 | 0.128 | 0.092 | ‒ | 0.475 | **0.191**** | ‒ | ‒ |
| **Lake Overstreet (LOV*x*FL)** | 20 | 10 | 1 | 2 | 0.200 | 0.154 | 0.018 | ‒ | 0.327 | **0.245**** | ‒ | ‒ |
| **Newport Sulphur Spring (NEW*x*FL)** | 21 | 10 | 4 | 4 | 0.822 | 0.072 | 0.16 | ‒ | 0.245 | **0.193**** | ‒ | ‒ |
| **Natural Bridge (NAT*x*FL)** | 22 | 9 | 2 | 3 | 0.639 | 0.126 | 0.068 | ‒ | 0.297 | **0.229**** | ‒ | ‒ |
| **Gambo Bayou (GAM*x*FL)** | 23 | 9 | 2 | 2 | 0.500 | 0.128 | 0.088 | ‒ | 0.285 | **0.222**** | ‒ | ‒ |
| **Tram Road (TRA*x*FL)** | 24 | 9 | 3 | 3 | 0.639 | 0.126 | 0.112 | ‒ | 0.294 | **0.214**** | ‒ | ‒ |
| **Wacissa River (WAC*x*FL)** | 25 | 10 | 4 | 4 | 0.800 | 0.076 | 0.14 | ‒ | 0.244 | **0.196**** | ‒ | ‒ |
| **Hillsborough River (HIR*x*FL)** | 32 | 11 | 1 | 2 | 0.182 | 0.144 | 0.016 | ‒ | 0.276 | **0.234**** | ‒ | ‒ |

Results are based on cyt*b* variation within regional groups, SAMOVA-inferred groups (also see Results), and populations. Numbers (Nos.) correspond to collection sites in Figure 1. Measures of DNA polymorphism including numbers of segregating sites (*S*) determining allelic richness (*h*; i.e. number of haplotypes); haplotype diversity (*Hd*) and its standard deviation (s.d.); and nucleotide diversity (π) multiplied by 100 are presented for **A)** lineages **B)** regional groups, **C)** SAMOVA groups and then **D)** populations whose respective sample sizes (N) met an N≥8 threshold criterion for defining ‘sufficient’ sampling (see Methods). Results of Fu’s *F*_S_, Ramos-Onsins and Rozas’ *R*_2_ and Fay and Wu’s *H* tests of neutrality based on coalescent simulations (10^4^ permutations) are also reported. McDonald–Kreitman test (‘MK test’) results correspond to the probability to reject neutrality by Fisher’s exact (two-tailed) test in McDonald and Kreitman [8]. Statistically significant results are presented in bold; **P<0.0001.

†MtDNA and nDNA sequences were named using these codes; *x*-variables in codes range 1-12 to represent each individual sequenced for this study.

‡No variation found in population.

**Table S6 Mitochondrial cyt*b* haplotype table and private alleles summary**

**Table S7 Nuclear *RPS7* haplotype table**

Tables S6 and S7 above are haplotype tables describing the geographical locations and frequencies (per collection site) for each gene sequenced in this study (except sequences, hence collections, omitted prior to analyses; see text, Additional file 1: Supplement S1 below). The cyt*b* haplotype data for *Heterandria formosa* are presented by locality in Table S6. The number in each cell indicates the number of individuals from a particular locality with a particular haplotype; empty cells denote zero. Table S7 presents the haplotype table for the nuclear *RPS7* data (for *H. formosa* and its putative sister taxon, Poeciliidae sp. from Coahuila, Mexico) in a similar format. Names of each locality are preceded by their numbers (corresponding to Figure 1, Table 1) and followed by parentheses containing corresponding specimen DNA codes (Table 1). One difference between the tables presented herein is that Table S6 also summarizes numbers of observed private alleles at each site, as well as the proportion of gene sequences from each site matching assumedly ‘derived’ network tip alleles (based on the cyt*b* network shown in Figure 7; as opposed to internal alleles), whereas Table S7 does not.

**Table S8 Results summary**

| **Predicted pattern** | **Method used** | **Hypotheses** |
| --- | --- | --- |
|  |  | **Expansion-contraction (22/33, 66.7%)** |
| **1. Relevant patterns of bioclimatic suitability during LGM (22-19 ka) to present** | paleoclimatic modeling, MAXENT | 2/2  (LGM, present-day models) |
| **2. Support for relevant gene flow barriers** | BARRIER and SAMOVA models‡ (independently tested by AMOVAs, Arlequin) | 2/2 |
| **3. Relevant patterns of isolation-by-distance** | Mantel tests of mtDNA and allozyme variation‡, GENALEX  (MtDNA: rangewide, FL;  allozymes: rangewide, WCP, ACP, FL, and within-refuge) | mtDNA: 2/2  allozymes: 2/5 (ACP and within-refuge allozyme results consistent with predictions) |
| **4. History of population bottleneck-expansions** | mtDNA neutrality tests, demographic modeling in Arlequin and inferred expansion timing  (WCP, ACP, and 4 SAMOVA-inferred groups) | 5/6  (SAMOVA group 2 inconsistent) |
| **5. Relevant patterns of genetic diversity** | spatial distributions of allozyme‡ diversity (*H*_e_) and private allelic richness (*h*_p_), nonparametric tests and linear models | 2/3  (overall *h*_p_ pattern inconsistent) |
| **6. Reciprocal monophyly of populations** | maximum-likelihood (GARLI) and Bayesian (BEAST) trees, parsimony networks (TCS), ‘Minimize Deep Coalescences’ tree (Mesquite), allozyme Neighbor-Joining tree (PAUP*) checked by PCoA (GENALEX) | 0/5 |
| **7. Relevant locations of basal populations** | phylogenies/parsimony networks | ~2/2  (phylogenetic conclusion tentative) |
| **8. Relevant locations of derived populations** | phylogenies/parsimony networks | 4/4  (putative refuge haplotypes mostly ancestral/interior; haplotypes from ‘outside’ putative refuge mostly tip/derived; most refuge haplotypes ancestral/basal in phylogenetic tree, Figure 6B) |
| **9. Relevant timing of (basal) population structure** | BEAST relaxed-clock coalescent-dating analyses | 0/1 |
| **9b. Relevant timing of Atlantic Coastal Plain population structure** | BEAST relaxed-clock coalescent-dating analyses | N/A |
| **10. Support for phylogeographical scenario** | coalescent simulations in Mesquite | 1/1 |

Results are presented following [9], with instances of statistical tests/analyses supporting each prediction followed by a slash then the total number of test/analysis instances [a sum of the number of individual tests or test comparisons supporting the hypothesis out of the total, excluding N/A (not applicable) cases, is given in parenthesis next to each hypothesis, along with the percentage of predictions supported]. Each instance of a prediction supported by our analyses is shaded gray.

‡For sufficiently sampled populations or regional groups (N≥8), except samples formed by pooling populations (Methods; Table 1).

**Supplement S1: Methods and results details**

## Sampling and laboratory methods

Initially, we inferred a 1-bp indel at a nuclear *RPS7* intron 1 site when the *Heterandria formosa* sample *RPS7* sequences were aligned against ‘Poeciliidae sp.’ sequences: some of the *H. formosa* samples were missing a ‘T’ at nucleotide 284 of the first intron, which corresponded to the 281^st^ position of our *RPS7* sequence alignment. This would have been of no consequence for our analyses of *H. formosa* populations anyway, because each *H. formosa* individual in the alignment possessed the indel. However, this appears to have been attributable to sequencing errors; in our final alignments, *H. formosa* and Poeciliidae sp. samples possess a ‘T’ at the 284^th^ position of the first intron. Other indels were inferred in the *H. formosa* and Poeciliidae sp. sequences when these were aligned against the ‘potential outgroups’ (Table S1). To ease potential concerns related to the introduction of the indels into the original nuclear alignment, it is worth noting that indels were first determined during the editing process using Sequencher 4.10 (Gene Codes Corp.), using the multiple sequence alignment algorithm in the software and checking the sequences by eye. We also translated sequences into amino acids to check for the presence of premature stop codons or other nonsense mutations, and while checking by eye we caught and eliminated an erroneous duplication (26 bp overlap between sequences). However, we created the alignments for our final analyses by running our data set through the multiple sequence alignment program MAFFT 6 [11] (starting from FASTA format); this algorithm inferred a similar pattern of nuclear indels at the same alignment positions relative to those we obtained in Sequencher (data not shown). Most of our coalescent-based analyses, including parametric tests in DnaSP 5.10 [10] and mismatch analyses, were not affected by insertion-deletions because they were based solely on our mtDNA matrices, which were unambiguous and had no indels. A mtDNA haplotype (allele) database was also used in gene tree-species tree simulations in Mesquite, which did not simulate indels.

## Ecological niche modeling

Methods for ecological niche-based modeling of species distributions (sometimes called ‘species distribution modeling’) provide several means of predicting the actual or potential distribution of a species, given some prior information on its occurrence (e.g. presence-only data, or presence-absence data based on known collection sites from museum records) as well as environmental predictor variables for those sites and the rest of the surface area you desire to model. To model the potential LIG (130-116 ka; data layers were from 140-120 ka, see text), LGM (22-19 ka) and present-day (0 ka) distributions of *Heterandria formosa*, we used the maximum entropy (maxent) approach as implemented in the software program MAXENT 3.3.3k [12], which is a powerful presence-only method. Maxent is a machine-learning technique that is highly useful because absence data, although useful for ecological niche models in practice, are unavailable for many species [12]. Maxent assumes that occurrence data points used in each analysis are from source, rather than sink, habitat or in other words the species realized niche [12]. We modeled *H. formosa* distribution based on a comprehensive set of 259 occurrences, spanning the entire species geographic range distribution. A map of the full set of occurrences used is shown in Figure S2 (above). This degree of sampling ensured good power for generating our model, as well as lower likelihood of running models based on sink habitat and accordingly greater likelihood of encountering a higher fraction of sites representative of the species realized niche. As described in the text, our environmental predictor variables were from the WorldClim data set (http://www.worldclim.org/; [7]). These represent annual trends and extremes derived from monthly temperature and rainfall data, and they have been shown repeatedly to be biologically meaningful (e.g. refs. in [12-14]). While conducting ecological niche modeling analyses, we conducted iterative MAXENT runs, using different combinations of our environmental data layers (Table S2, above) to determine the most suitable variables to include in our final model(s). The results of one set of current/paleo-reprojection runs using the six most important bioclimatic variables (see Tables S3-S4) yielded nearly identical results to the full models including all 19 layers, indicating our analyses were not confounded by model over-fitting effects (e.g. [15], refs. therein). Therefore, we incorporated all 19 layers into our final analyses regardless of correlations among variables, as iterative analysis showed these correlations did not lead to spurious results. We discuss this here to provide an example of one step in the iterative process by which we arrived at a desirable model with good predictive power, free of effects of confounding factors.

To provide a picture of the possible configurations of drainage basins during colder/drier conditions and more than 100 m lower sea levels of the LGM, we consulted available bathymetric models. One colleague of ours, Peter J. Unmack, recently developed a paleo-bathymetric rivers GIS layer (http://peter.unmack.net/gis/sea_level/) that visualizes predicted river paths over a −135 m (relative to present-day sea level) continental shelf contour, worldwide. Unmack et al.’s [16] paper contains details on how a similar dataset was generated for the continental shelf areas surrounding Australia. We present the results of Unmack’s model, restricted to continental shelf areas near our study area (Gulf of Mexico, northwest Atlantic Ocean), in Figures 1, 2, and also S1 (above; used with permission from P. J. Unmack). We refer to this layer as an external source of paleoenvironmental data forming a basis for broad biogeographical prediction and interpretation, e.g. discussed in the main text. In pilot analyses, we also incorporated masks based on joining this paleo-drainage dataset with a GIS dataset for modern river paths into ecological niche models similar to those reported herein. This gave models similar to those in Figure 3, but showed that incorporating drainage information, although producing a potentially more accurate model (accounting for the inhospitable matrix of terrestrial habitat between river stems), did not qualitatively alter results. Moreover, the paleo-bathymetric + modern rivers layer made 0% contribution to model prediction and therefore was otherwise superfluous (unpublished data).

Applications of maxent to freshwater aquatic taxa in phylogeography studies clearly remain in their infancy. Indeed, due to limitations of geospatial data sets currently developed (i.e. being mostly from climate circulation models), our niche models have not been able to capture fine-scale variation in marsh and wetland habitats that these fishes typically inhabit. As mentioned in the text, our models have some potential biases such as this that we were not able to account for, and more detailed modeling attempts with more high-resolution data layers will be necessary to assess that bias and develop better models for coastal taxa such as *H. formosa*. However, this might mean that an on-the-ground effort by biologists is needed to collect data and develop GIS data layers at sufficient and relevant spatial scales. With that said, it has long been recognized that spatially modeling species responses to ecological/environmental phenomena, and thus species distribution modeling (e.g. ecological niche models), at very fine scales is usually problematic due to lack of suitable environmental-climatic data [14]. Thus, this problem is not unique to *Heterandria formosa* but presents a major challenge for modeling most species distributions on Earth.

One key issue for freshwater species ecological niche modeling is that, as encountered in our study, workers are limited in the number and quality of data layers available across multiple time slices, thus in predicting paleodistributions across multiple points in the past. Currently, it is possible to use high-resolution bioclimatic variables from the LIG-present. However, paleoenvironmental data on drainage basin positions are not available in many cases, e.g. for the LIG. Thus, using a paleo-drainage/environmental approach similar, but much more comprehensive, to that above will in the future present a critical means of integrating information across spatial and temporal scales. Only once such resources are in-hand will more realistic biogeographical hypotheses be derived across wider timespans from geospatial data.

Despite these issues, we think it reasonable to conclude that our ecological niche modeling and LGM paleodistribution models of *H. formosa* have captured something meaningful about the biology and historical ecology of this organism, within current modeling constraints. Particularly, this is because the data we were able to employ captures information about critical environmental variables influencing freshwater fishes, we used a high-performance algorithm, the iterative process we took ensured models were improved and tested for performance/fit to the data, and the sample of occurrences we were able employ in our analyses was comprehensive. Moreover, although finer-scale, higher-resolution data (e.g. on marsh habitats) might have improved our models, it is widely accepted that the variables our models relied on most (climatic variables) are appropriate at the scales of our actual analysis (the global- to meso-scales, i.e. of subcontinental physiographic provinces; well above the local scale) and likely affect species distributions at those scales [14].

## Population structure and genetic diversity

SAMOVA groups shown in Figure 1 included the collection sites listed in Table S1 (other sites were either pooled with these to obtain sufficient sample sizes for analysis, or not considered). These SAMOVA-inferred groups were used in further demographic analyses (e.g. mismatch distribution analyses discussed below and in the text).

We based our analyses of molecular variance (AMOVA) on populations with comparable and sufficient sampling (N≥8), and only used/ran models with more than two comparisons. Arlequin AMOVA results are reported as percentages representing hierarchical partitioning of cyt*b* diversity across levels. AMOVAs are based on Φ-statistics, whose values range from 0, indicating no genetic structure, to a maximum of 1, indicating complete isolation. Φ_CT_ is the correlation of random haplotypes within a group relative to the whole dataset (among groups); as noted in the text, this statistic reflects the proportion of total genetic variance among geographically defined groups of populations. Φ_SC_ is the correlation of the diversity of random haplotypes within populations relative to random pairs from the same group of populations (within regions). Φ_ST_ is the correlation of random haplotypes within populations relative to random pairs drawn from the entire dataset.

Results of AMOVA models independently testing the best BARRIER and SAMOVA grouping schemes are presented in full here. The 4-group SAMOVA model was strongly supported by AMOVA, with P<0.0001 for among-group differentiation (Φ_CT_=0.72); thus AMOVA confirmed distinctiveness of SAMOVA groups. During the SAMOVA-group comparison, 72.02%, 9.97%, and 18.01% of genetic variation were partitioned respectively among groups, among populations within groups, and within populations. Other Φ-statistic values (levels of differentiation) were also significant, consistent with significant spatial (phylogeographic) structure overall (Φ_SC_=0.36, P<0.0001) and among populations (Φ_ST_=0.82, P<0.0001). The 4-group model testing the BARRIER grouping scheme was also strongly supported by AMOVA analysis (Φ_CT_=0.73, P<0.0001). In this comparison, 73.43%, 9.23%, and 17.34% of genetic variation were partitioned respectively among groups, among populations within groups, and within populations. Other Φ-statistic values were also significant: Φ_SC_=0.35 (P<0.0001) and Φ_ST_=0.83 (P<0.0001). Variance partitioning (%) in the BARRIER-group comparison were similar to those for the SAMOVA-group comparison across hierarchical AMOVA levels. We interpreted this as indicating that geographical regions containing genetic barriers identified by both methods are potentially capable of reducing regional gene flow to a similar degree. This would make sense given most barriers were inferred in the same region, between the Apalachicola River and the east end of Apalachee Bay.

Mantel tests for isolation-by-distance based on the mtDNA data were performed rangewide and within the FL regional group using relevant collections (mostly that met a sampling threshold of N≥8; Table 1), and the results are given in full in the main text. Mantel tests for other groupings/levels not listed here were essentially not possible, e.g. due to limited within-site sampling (N<8), or irrelevant. Mantel tests were based on the normalized Mantel coefficient (*r*; similar to Pearson’s *r*, but not to be confused with the raggedness statistic) and p-values (right-tailed) for the observed *r* between the two matrices were based on 10^4^ permutations of the *F*_ST_ data in GENALEX. We observed no significant patterns of mtDNA isolation-by-distance, and each of non-significant relationship was confirmed by linear regression analyses in PAST. These results were consistent with predictions of both of our hypotheses.

We conducted similar Mantel tests during our re-analysis of Baer’s [17] *Heterandria formosa* allozyme dataset, described in [17] and the text. Allozyme Mantel test results were based on unbiased Nei’s *D* genetic distances (*D*_Nei_). Full results from GENALEX were as follows: rangewide (see text); WCP (N=6 populations), *r*=0.776, P=0.042; FL (N=22 populations), *r*=0.203, P=0.014; ACP (see text); and within-refuge (Figure 3B; N=7 populations), *r*=0.360, P=0.014. Results of linear regression model analyses for these groups in PAST supported the Mantel test results and were as follows: rangewide regression (see text); WCP regression R^2^=0.603, *t*=4.448, P=0.0007; FL regression R^2^=0.041, *t*=3.141, P=0.002; ACP (see text); within-refuge regression R^2^=0.498, *t*=2.815, and P=0.022. These results were essentially opposite to the mtDNA-based Mantel results, with significant isolation-by-distance across much of the species range, except within the ACP region. However, significant IBD within the putative refugial area inferred by the niche models was consistent with the mtDNA results. Overall, these results appear to favor isolation-by-distance within the putative refuge, the expected pattern under a scenario of expansion-contraction; however, the inferred presence of isolation-by-distance throughout the remainder of the range was surprising and is discussed further in the text.

## Historical demography

Some of our mtDNA neutrality test results were beyond the scope of that presented in the main text and Table S1 (above). The full details of Fay and Wu’s *H* test conducted at the level of each regional group are as follows: WCP mean *H*=-0.0129 [-5.0714, 2.214], P=0.35; FL mean *H=*0.0544 [-13.412, 5.0297], P=0.32; ACP mean *H*=0.00512 [-8.250, 3.528], P=0.338. The details for 95% confidence intervals and P-values for Fay and Wu’s *H* test conducted at the level of clades are as follows: subclade ‘a’ [-11.777, 4.355], P=0.317. The details of the estimated 95% confidence intervals and P-values for Fay and Wu’s *H* test for each SAMOVA group are as follows: SAMOVA group 1, [-9.689, 3.733], P=0.34; SAMOVA group 2, [-7.151, 2.632], P=0.33; SAMOVA group 3, [-5.082, 1.907], P=0.33; SAMOVA group 4, [-5.498, 2.123], P=0.32.

We conducted mismatch analyses in Arlequin. We tested the goodness-of-fit of the data to mismatch distributions, and the P-values for the tests were derived by calculating Harpending’s raggedness index (*r* [18]) as the test statistic for the observed data and comparing it to *r* calculated from 1000 parametric bootstrap simulations of the original data. Harpending’s [18] *r* measures the smoothness of the observed pairwise differences distribution and can be taken as the significance level by which the hypothesis of no ancient expansion is rejected. Low *r-*values are expected for expanding populations and indicate good fit between mismatch distributions and the data. Higher *r*-values are expected for non-expanding populations that have been constant (experienced stable mutation parameters through time) and indicate more probability of rejecting ancient expansion [18]. Results were considered significant at the α=0.05 level, and we failed to reject expansion models in all cases. The resulting P-values from Arlequin represent the probability of the expected *r* (calculated from the simulations) being greater than or equal to the observed *r*. The P-values for *r* were as follows: WCP, P=0.09; ACP, P=0.36; SAMOVA group 1, P=0.65; SAMOVA group 2, P=0.10; SAMOVA group 3, P=0.19; SAMOVA group 4, P=0.57.

Unfortunately, *r* has low power to detect population expansion. As a result, we also tested whether a null hypothesis of population stasis could be rejected in favor of non-neutrality and expansion using parametric tests of other statistics, Fu’s *F*_S_ and *R*_2_, which are more sensitive to past population expansions. In all cases, *R*_2_ and mismatch (*r*) supported expansions within regional groups (Figure 1; Table 1) and SAMOVA groups in Figure 1.

## Phylogenetic relationships and coalescent-dating analyses

As mentioned in the main text, we selected DNA substitution models that were most appropriate for each of our molecular DNA datasets using the decision theory algorithm implemented in DT-ModSel [19]. This method selects models that are simpler and that result in more accurate branch lengths than those chosen by conventional likelihood-ratio test statistic-based methods [19]. The appropriate models were used during our molecular DNA sequence analyses.

During multilocus phylogenetic maximum likelihood analyses ran in GARLI 0.97 [20], the gene datasets we used contained sequences from individuals representing ‘subsamples’ of the entire collection of specimens obtained for this study, including cyt*b* and *RPS7* sequences for each of 17 *H. formosa* and 2 Poeciliidae sp. samples. Thus the alignment included 38 *H. formosa* and Poeciliidae sp. sequences, plus 72 sequences for 39 additional ‘potential outgroups’ listed in Table S1 above, which represented 23 additional lineages. The total length of the multilocus DNA alignment, for which we had data from a 58 tip taxa, was 2041 bp (1140 bp cyt*b*, plus 876 bp *RPS7* that was 901 bp long after alignment against potential outgroup sequences). We specified separate models for different partitions of each gene in this alignment. Specifically, we partitioned the cyt*b* data by codon position, and appropriate models of evolution of each cyt*b* partition-subsets were as follows: cyt*b* codon positions 1+2, TrN+Γ+*I*; codon position 3, GTR+Γ+*I*. In addition, the best model selected for the *RPS7* gene dataset was K80. We did not assign separate models of evolution for different *RPS7* codons. DT-ModSel selected similar best-fit evolutionary models for our *H. formosa* cyt*b* haplotype dataset (N=47; using haplotypes in Table S6); for this dataset, the best models were as follows: cyt*b* codon positions 1+2, TrN+*I*; codon position 3, TrN. We analyzed this haplotype alignment separately in GARLI to generate maximum-likelihood ‘best’ trees, which we used to test our hypotheses using coalescent simulations. We used the resulting haplotype tree as the starting tree for our ‘Minimize Deep Coalescences’ species tree calculation.

The phylogenetic analyses we conducted on our multilocus DNA sequence database (maximum likelihood phylogenetic analysis, coalescent relaxed-clock dating analyses), and the McDonald–Kreitman [8] tests for selective neutrality (see text), were our only analyses in which we specified outgroup taxa. In each of these analyses, 2 samples of Poeciliidae sp. (mentioned above; each unique alleles at cyt*b*; Table S6) served as the outgroup in our initial analyses. As noted in the text, we iteratively assessed the impact of using other potential outgroup taxa listed in Table S1 on results. Regarding the McDonald–Kreitman tests, using different potential outgroups did not qualitatively alter the results of the tests. Thus, we report results from the initial tests. In the case of our phylogenetic analyses, outgroups impacted divergence time estimates (presumably leading to improved estimates) because they allowed calibration points. However, using different outgroups did not change ingroup results significantly in any case (JCB, unpublished data). In other words, outgroup sampling did not alter the pattern of phylogeographical relationships recovered within *H. formosa*. GARLI or BEAST runs using different outgroups also did not recover any other lineage as sister to *H. formosa* except Poeciliidae sp. (as long as Poeciliidae sp. was included in the alignment). On this point, it is worth noting that including many potential outgroups from other Poeciliidae lineages in our phylogenetic alignments and leaving *H. formosa* free to move throughout the tree (not constrained to be monophyletic or sister to any one taxon) permitted conducting an outgroup analysis, where we allowed the data to tell us the most likely outgroup taxon rather than simply assuming *a priori* that Poeciliidae sp. was the outgroup. We recovered Poeciliidae sp. as sister to *H. formosa* with high nodal support. Thus, we have clearly demonstrated that Poeciliidae sp. samples used in this study are more closely related to *H. formosa* than any other species based on mtDNA and nDNA sequence variation.

Our BEAST analyses are adequately described in the main text. However, aside from other results, we report likelihoods of some of our BEAST models in the text. We here remind readers that likelihood scores from any particular computer program mentioned here or in the text should not be assumed to be appropriate to compare with likelihood scores from other programs.

**Figure S3 ‘Best’ maximum-likelihood gene tree topology inferred from GARLI analysis of cyt*b* and *RPS7* sequence data**

This tree presents the results of an outgroup analysis (see Methods) conducted in GARLI through maximum-likelihood phylogenetic analysis on our *H. formosa* and Poeciliidae sp. samples, plus additional potential outgroup taxa (Table S1). Numbers by each node are bootstrap support values >50 (based on 500 bootstrap pseudoreplicates), although we only consider values ≥70 to provide strong nodal support.

## Hypothesis testing and statistical phylogeography

## *Incorporating BEAST results into the simulations*

During our coalescent simulations, tree depths for hypothetical population trees representing our hypotheses were set based on the estimated *t*_MRCA_ values we obtained during coalescent-dating analyses (genetic simulations) in BEAST 1.74 [21]. The BEAST analyses were run on the multilocus datasets discussed above (N=58 taxa, cyt*b* and *RPS7* sequences) and required evolutionary models to be specified. We created the input (.xml) files for these analyses in the BEAST utility program BEAUti. We divided the dataset into identical codon positions compared to those used in our GARLI analyses above, which we had already run in DT-ModSel, so (obviously) we applied the same best-fit site models during our BEAST runs. Again, the best-fit models were as follows: cyt*b* codon positions 1+2, TrN+Γ+*I*; cyt*b* codon position 3, GTR+Γ+*I*; *RPS7* gene dataset, K80. Our BEAST analysis employed the uncorrelated lognormal (ULN) relaxed-clock model. As a result, no BEAST models in this study made the assumption of constant mutation rate over time (evolutionary rate-constancy, or ‘clock-likeness’), although we did assume constant coalescent population sizes (demographic models) during analysis. BEAST, like other similar coalescent-genealogy sampling software programs, assumes random sampling, no selection, random mating within subpopulations, no recombination, stable subpopulation structuring over time, and the same copy number for all loci [21,22]. We were justified in using a relaxed-clock model based on results of pilot runs conducted in BEAST testing the assumption of clock-likeness for our data (using the ULN model and MCMC=10^7^ steps, burn-in=10^6^), based on the standard deviation of the relaxed clock (‘ucld.stdev’) parameter. By this test, marginal distributions of the ucld.stdev parameter including zero indicate the molecular clock hypothesis cannot be statistically rejected, while ucld.stdev distributions whose lower confidence intervals fall above zero or much greater than 1.0 indicate substantial among-lineage rate heterogeneity, given the data. In our pilot runs, marginal ucld.stdev distributions clumped above zero, statistically rejecting the hypothesis of clock-like data based on the 95% highest density of the posterior; e.g. from one run, we obtained mean ucld.stdev (for cyt*b* data block)=0.534, with 95% confidence intervals=[0.328, 0.753], ESS=2003.68.

BEAST is a Bayesian coalescent sampler that estimates historical demographic parameters, e.g. Bayesian skylines, while simultaneously incorporating error in the genealogy and the coalescent [21,23]. As a result, our coalescent divergence-dating results are partly robust to potentially confounding effects of coalescent stochasticity, although inferences could probably have been improved if more gene sampling at unlinked loci and additional intraspecific calibration points had been available to us. However, intraspecific calibration points (e.g. heterochronous samples, ancient DNA, microfossils, etc.) are extremely rare in phylogeography studies, thus this is a problem for most taxa, and not an issue unique to *H. formosa*.

## *Incorporating MIGRATE-N and DnaSP results into the simulations*

In addition to genealogical depths of simulations, another key parameter in coalescent simulations is effective breeding population size, *N*_e_. We based our coalescent simulations on *N*_e_ values estimated from empirical population size parameter (*θ*) estimates calculated in the programs DnaSP [10] and MIGRATE-N 3.1.3 [24], as described in the main text, for our four SAMOVA groups (shown in Figure 1, listed in Table S1 above). In DnaSP, we calculated Watterson’s estimator *θ*_W_ (per site) and its standard deviation based on the number of segregating sites (*S*; see DnaSP manual for further references and discussion of this parameter).

However, we also estimated *N*_e_ from empirical population size parameter (*θ*) estimates obtained using statistical phylogeography, sampling over many genealogies, in MIGRATE-N. Our input files consisted of the full *H. formosa* cyt*b* dataset, subdivided into each of the four SAMOVA groups, which were paired in each input file. By conducting pairwise analyses, we were able to ensure better (more likely, and faster) chain convergence, and because modeling two populations at a time is below the limit (approximate maximum is ~5 populations; MIGRATE-N does not handle more than 3-5 populations well) within which MIGRATE-N performs well (was ‘designed for’; sometimes with >5 populations a run may essentially never converge or even finish). Thus, we were able to use ‘full models’ estimating all parameters, and custom stepping-stone models were not necessary to constrain run times or produce more biologically meaningful results, e.g. among distant populations. We found convergence was reached and results were adequate. This method of using pairwise comparison among group also kept the number of parameters lower than running a single model including data from all four populations.

MIGRATE-N assumes the standard finite sites model of DNA/RNA evolution. In addition, the program assumes random sampling, no selection, random mating within subpopulations, no recombination, stable subpopulation structure and constant population size over time, identical copy number of all loci, and that the samples were taken contemporaneously. Our results suggest our cyt*b* data are consistent with some of these assumptions, particularly no selection on the mtDNA genome. MIGRATE-N can be run with constant mutation rates, or with rates estimated from a prior distribution; however, the program assumes that the mutation rate per locus is constant. A benefit of MIGRATE-N over other coalescent samplers is that it can analyze more than two to three populations; however, the program does not perform well with many populations plus many loci (although multiple loci give better results themselves). It also gives a range of different outputs, including its own version of the Bayesian skyline plot, likelihood surfaces, and improved approximations of likelihoods which can be used to compute Bayes factors for model comparisons (when likelihood inference is used, MIGRATE-N can conduct likelihood ratio tests and model selection based on AIC scores).

MIGRATE-N estimates Θ as well as two versions of migration rates, the mutation-scaled migration rate (*M*=*m/u*) as well as the effective number of migrants per generation between groups/populations (*Nm*). The different migration parameter estimates must be obtained through separate types runs, with ‘M’ runs (setting: “use-M=YES”) being used to calculate *θ* plus *M*, and ‘Nm’ runs (setting: “use-M=NO”) needed to obtain *θ* estimates and direct *Nm* estimates. We obtained parameter estimates by running MIGRATE-N under a Bayesian inference algorithm based on the standard Metropolis-Hastings (accept/reject) algorithm during MCMC searches of the main parameters. We set MIGRATE-N to run a single long (MCMC search) chain (3 × 10^8^ steps) sampled every 20 steps (or 1.5 × 10^7^ samples); 1-10 million steps discarded as ‘burn-in’; with flat *θ* [0.0, 0.1] and *M* [0.0, 1000.0; mean=100; δ=50] priors covering published values for most vertebrates; and with a uniform mutation prior consistent with rates of vertebrate mtDNA evolution, and the ‘fish rate’ reported in the text. To confirm MCMC chain convergence on similar values, we ran the program multiple times, and values used in our simulations and reported herein are based on three replicate runs. We ran three sets of final MIGRATE-N runs for each SAMOVA group pair, under both ‘use-M’ run options, and thus we were able to respectively estimate *M* as well *Nm*, from separate analyses. We converted mean *θ* estimates for each group to *N*_e_ as described in the text.

In terms of prior settings, here is an example of the code we used to make one set of basic Bayesian priors that we modeled (note: we specified a mutation rate prior, although MIGRATE-N doesn’t actually use this information for most analyses):

“bayes-priors= THETA UNIFORMPRIOR: 0.000000 0.1 0.0500000

bayes-priors= MIG WINDOWEXP: 0.000000 100.000000 1000.000000 50.000000

bayes-priors= RATE UNIFORMPRIOR: 0.010000 100.000000 5.000000.”

Uniform priors are less desirable because all values are probably not actually equally likely, as such priors assume. The exponential window prior has superior performance to the uniform prior. It is also important, under Bayesian inference in MIGRATE-N, to set priors to overshoot the likely actual value for the data; so setting broad uniform priors like ours ensures that searches that are sufficiently long will converge on a smaller value than the upper bound (i.e. not get stuck or pile up at the upper bound).

A range of *N*_ef_ estimates (including means derived from the *θ* estimates in DnaSP and MIGRATE-N) used in the simulations is presented in the main text. The full results of the DnaSP *θ*_W_ estimates are provided in Table S1 above. Here, we present full results of the Θ and *N*_ef_ estimates from MIGRATE-N. Results are shown in Table S9 below, by SAMOVA group with 95% confidence intervals from the Bayesian posterior distribution in brackets (presenting results from ‘M’ runs only).

**Table S9 MIGRATE-N population mutation rate and effective size results summary**

| **SAMOVA group** | **mean Θ** | **estimated mean *N*_ef_** |
| --- | --- | --- |
| **group 1** | 0.00226 [0.000, 0.00480] | 158707.87 |
| **group 2** | 0.00360 [0.00080, 0.00620] | 252808.99 |
| **group 3** | 0.00106 [0.000, 0.00300] | 74438.20 |
| **group 4** | 0.00169 [0.000, 0.00400] | 118679.77 |
| **Overall *N*_ef_ (sum)** | - | 604634.83 |

We note here that, in other analyses aside from those presented in the main text, we used the output of our MIGRATE-N models to calculate migration probability per individual per generation [e.g. (mean *N*_ef_*m*, recipient population)/(mean *N*_e_, source population)], e.g. [25]. We performed additional simulations wherein we incorporated this probability by specifying bursts of migration during the last 66,000 generations—equivalent to migration since the onset of the LGM and -120 m sea levels. We allowed the migration probability estimate we obtained (e.g., based on one population pair and averaged across both populations, migration probability= 4.0825 × 10^-10^/individual/generation) to be the probability of migration of any allele between any two of the 40 populations in the areas in our hypothetical population tree models, at any point in time since the LGM. This method was more realistic, given that assuming (in our null expansion-contraction model) that populations have experienced no migration during their expansion from a south Florida refugium is probably unrealistic. However, these additional simulations produced results that were qualitatively identical to the results presented in the main text for simulations not accounting for potential random migration. Moreover, including migration had very little quantitative effect on the results. This might reflect the fact that the inferred migration probabilities were very low, or that the short time span of the burst produced very little migration among populations in the simulation. However, as a result, we only report our findings for the more simplistic models, without migration.

## *Hypothetical and observed gene trees and Mesquite*

In the main text, we provide a description of the hypothetical population trees and gene trees used in our coalescent simulations. Here, we provide additional details. As noted in the text, branch-lengths units are time in generations (scale not exact) and node ages indicate timing of divergence/colonization. For simulations, all tree depths (*t*_Total_) were set to *t*=1.247 Ma (Early Pleistocene), the *H. formosa* *t*_MRCA_ estimate from BEAST.

The ‘fragmented ancestor’ representation of the null model representing the expansion-contraction hypothesis included a long root branch dating back to the species *t*_MRCA_, then a 90% reduction in ancestral *N*_e_ during the LGM (22-19 ka; with the date of the reduction being *t*1=22 ka, and the lengths of tip branches/populations diversifying after recovery being set to *t*2=tip branches≈15-0 ka, including Holocene. Gene trees were simulated within this topology. The null expansion-contraction hypothesis was analyzed against two alternative hypotheses, including a vicariance-northeast colonization model, and a ‘four-refugia’ model. The ‘vicariance’ component of vicariance-northeast colonization was modeled as a basal split (initial interpopulation divergence) just west of the Apalachicola River. So, the population tree grouped all WCP samples into one population-lineage, and all samples east of the Apalachicola River into another population-lineage (internal branch). This initial vicariance event was followed by ACP colonization during the LGM, thus ACP populations were grouped in a shallow polytomy branching from the St. Johns River population (tip branch) at ~19-16 ka, and all tips (ACP) diversifying into the Atlantic seaboard from this event were set to a length of *t*2=15-0 ka. The four-refugia model was similar to the vicariance-northeast colonization model, but with four internal branches/population-lineages, instead of two, representing the diversification of *H. formosa* in four separate Pleistocene refugia corresponding to the positions of the four SAMOVA groups (Figure 1, Table S1) at the time of the LGM. Subsequently, the fragmentation of the four *H. formosa* refugial populations (SAMOVA groups 1-4; which might also be interpreted as spatial expansion without demographic expansion) was modeled by allowing each subpopulation to radiate out from its respective population-lineage post-LGM since *t*2=15-0 ka, becoming its own isolated population. It did not matter if the tip branches were modeled using the *t*2 just mentioned, which was identical to that used in the other models, or a *t*2=19-0 ka, immediately following the LGM; both models produced identical results. Branch widths were scaled according to proportions of overall *N*_e_ (=ancestral population; presented in the text) represented by each refugial population, which sum to overall *N*_e_ at each time point. We list these scaled widths here. The proportions of overall *N*_e_ for each refugial population/internal branch were as follows. For the null expansion-contraction model, overall *N*_e_ was simply subdivided evenly across all tip populations (tip width=overall *N*_e_/*n*_tips_). For vicariance-northeast colonization, the total proportion of the WCP lineage was set to 0.1631, evenly divided among tip populations; and the total proportion of the east-of-Apalachicola R. lineage was set to 0.8369, evenly divided among tip populations. The proportion of overall *N*_e_ of the single St. Johns River source population was ~0.0200-0.0400, whereas that of all the five diversifying ACP populations extending from it was (in total) ~0.1396 (thus the branch leading to the diversification point had width=~0.0200+0.1396). For the four-refugia model, the total proportions of each of the SAMOVA-group lineages were set to (group) 1=0.2625, 2=0.4181, 3=0.1231, and 4=0.1963, each evenly divided among tip populations. Root branches were (obviously) set to proportions of 1.0000 in each hypothetical population tree used in the simulations.

During our coalescent simulations, we used the fit of our ‘best’ maximum-likelihood gene tree to conduct hypotheses testing, as discussed in the text. When compared with a Minimize Deep Coalescences tree estimated from the same gene tree, it is clear that our maximum-likelihood topology is subject to incomplete lineage sorting. Thus coalescent simulations are an ideal means of evaluating this tree, and the species population history. The Minimize Deep Coalescences tree, itself, was generated using Maddison and Knowles’ [26] method implemented in Mesquite 2.73 [27]. This method finds the re-rooting of a given tree or trees that minimizes the deep coalescence cost, and it has been shown to increase probability of obtaining accurate population trees using even a single locus [28]. To implement this method, we opened our ‘best’ maximum-likelihood tree in Mesquite and then used the tree search function to find the population tree minimizing the number of deep coalescences (nDC; described in the main text) using subtree pruning and regrafting branch swapping based on parsimony. In a recent study of poison dart frogs, Wang and Shaffer [29] also used a similar method. By including this Minimize Deep Coalescences tree in our study and also conducting coalescent simulations using the maximum-likelihood topology, we were able to estimate the population tree given the data (assuming a regional population structure of a fragmented ancestor with population evolving simultaneously, free of assumptions about *N*_e_) and, second, we could look at the results of two methods to assess the influence of incomplete lineage sorting.

Whereas it might seem to follow that it would have been best to base our simulations on the Minimize Deep Coalescences species tree, this would not be the best methods because the simulations model the number of deep coalescences; thus, using a test topology that has had its deep coalescent events altered using the MDC method would have biased the results towards failing to reject the null Fragmented Ancestor model, even when the observed maximum-likelihood topology could have evolved within a given population tree i.e. hypothesis (Type II error; JCB, unpublished data).

## *Number of simulations*

We ran two sets of simulations within the population trees discussed above, based on *θ* estimates derived from DnaSP and MIGRATE-N results discussed above and in the main text. Thus we obtained 1000 gene genealogies simulated at each of the overall *N*_e_ (*N*_ef_) estimates reported in the text. Results were identical, decisively rejecting each of the alternative models in favor of the null expansion-contraction model.

**References**

1. Hrbek T, Seekinger J, Meyer A: **A phylogenetic and biogeographic perspective on the evolution of poeciliid fishes.** *Mol Phylogenet Evol* 2007, **43**(3):986-998.

2. Mateos M, Sanjur OI, Vrijenhoek RC: **Historical biogeography of the livebearing fish genus Poeciliopsis (Poeciliidae: Cyprinodontiformes).** *Evolution* 2002, **56**(5):972-984.

3. Agoretta A, Domínguez-Domínguez O, Reina RG, Miranda R, Bermingham E, Doadrio I: **Phylogenetic relationships and biogeography of *Pseudoxiphophorus* (Teleostei: Poeciliidae) based on mitochondrial and nuclear genes.** *Mol Phylogenet Evol* 2013, **66**(2013):80-90.

4. Miya M, Takeshima H, Endo H, Ishiguro NB, Inoue JG, Mukai T, Satoh TP, Yamaguchi M, Kawaguchi A, Mabuchi K, Shirai SM, Nishida M: **Major patterns of higher teleostean phylogenies: a new perspective based on 100 complete mitochondrial DNA sequences.** *Mol Phylogenet Evol* 2003, **26**(1):121-138.

5. Langerhas RB, Gifford ME, Domínguez-Domínguez O, Garcia-Bedoya D, Dewitt TJ: ***Gambusia quadruncus* (Cyprinodontiformes: Poeciliidae): a new species of mosquitofish from east-central Mexico.** *J Fish Biol* 2012, **81**(5):1514-1539.

6. Doadrio I, Perea S, Alcaraz L, Hernandez N: **Molecular phylogeny and biogeography of the Cuban genus *Girardinus* Poey, 1854 and relationships within the tribe Girardinini (Actinopterygii, Poeciliidae).** *Mol Phylogenet Evol* 2009, **50**(2009):16-30.

7. Phillips SJ, Anderson RP, Schapire RE: **Maximum entropy modeling of species geographic distributions**. *Ecol Model* 2006, **190**(3-4):231-259.

8. Mcdonald JH, Kreitman M: **Adaptive protein evolution at the *Adh* locus in *Drosophila***. *Nature* 1991, **351**(6328):652-654.

9. Solomon SE, Bacci M, Martins J, Vinha GG, Mueller UG: **Paleodistributions and Comparative Molecular Phylogeography of Leafcutter Ants (*Atta* spp.) Provide New Insight into the Origins of Amazonian Diversity**. *PLoS One* 2008, **3**(7).

10. Librado P, Rozas J: **DnaSP v5: a software for comprehensive analysis of DNA polymorphism data**. *Bioinformatics* 2009, **25**(11):1451-1452.

11. Katoh K, Misawa K, Kuma K, Miyata T: **MAFFT: a novel method for rapid multiple sequence alignment based on fast Fourier transform**. *Nucleic Acids Res* 2002, **30**(14):3059-3066.

12. Hijmans RJ, Cameron SE, Parra JL, Jones PG, Jarvis A: **Very high resolution interpolated climate surfaces for global land areas**. *Int J Climatol* 2005, **25**(15):1965-1978.

13. Waltari E, Hijmans RJ, Peterson AT, Nyari AS, Perkins SL, Guralnick RP: **Locating Pleistocene refugia: comparing phylogeographic and ecological niche model predictions**. *Plos One* 2007, **2**(7).

14. Mackey BG, Lindenmayer DB: **Towards a hierarchical framework for modelling the spatial distribution of animals**. *J Biogeogr* 2001, **28**(9):1147-1166.

15. Gür H: **The effects of the Late Quaternary glacial-interglacial cycles on Anatolian ground squirrels: range expansion during the glacial periods?** *Biol J Linn Soc* 2013, **109**:19-32.

16. Unmack PJ, Bagley JC, Adams M, Hammer MP, Johnson JB: **Molecular phylogeny and phylogeography of the Australian freshwater fish genus *Galaxiella*, with an emphasis on dwarf galaxias (*G. pusilla*)**. *PLoS One* 2012, **7**(6):e38433.

17. Baer CE: **Species-wide population structure in a southeastern U.S. freshwater fish, *Heterandria formosa*: gene flow and biogeography**. *Evolution* 1998, **52**(1):183-193.

18. Harpending HC: **Signature of ancient population growth in a low-resolution mitochondrial DNA mismatch distribution**. *Hum Biol* 1994, **66**(4):591-600.

19. Minin V, Abdo Z, Joyce P, Sullivan J: **Performance-based selection of likelihood models for phylogeny estimation**. *Syst Biol* 2003, **52**(5):674-683.

20. Zwickl DJ: **Genetic algorithm approaches for the phylogenetic analysis of large biological sequence datasets under the maximum likelihood criterion**. Austin, TX: The University of Texas; 2006.

21. Drummond AJ, Rambaut A: **BEAST: Bayesian evolutionary analysis by sampling trees**. *Bmc Evol Biol* 2007, **7**.

22. Kuhner MK: **Coalescent genealogy samplers: windows into population history**. *Trends Ecol Evol* 2009, **24**(2):86-93.

23. Drummond AJ, Rambaut A, Shapiro B, Pybus OG: **Bayesian coalescent inference of past population dynamics from molecular sequences**. *Mol Biol Evol* 2005, **22**(5):1185-1192.

24. Beerli P, Felsenstein J: **Maximum likelihood estimation of a migration matrix and effective population sizes in n subpopulations by using a coalescent approach**. *P Natl Acad Sci USA* 2001, **98**(8):4563-4568.

25. Shepard DB, Burbrink FT: **Phylogeographic and demographic effects of Pleistocene climatic fluctuations in a montane salamander, Plethodon fourchensis**. *Mol Ecol* 2009, **18**(10):2243-2262.

26. Maddison WP, Knowles LL: **Inferring phylogeny despite incomplete lineage sorting**. *Syst Biol* 2006, **55**(1):21-30.

27. Maddison WP, Maddison DR: **Mesquite: a modular system for evolutionary analysis.** In*.*, 2.73 edn; 2010.

28. Knowles LL, Carstens BC: **Estimating a geographically explicit model of population divergence**. *Evolution* 2007, **61**(3):477-493.

29. Wang IJ, Shaffer HB: **Rapid color evolution in an aposematic species: a phylogenetic analysis of color variation in the strikingly polymorphic strawberry poison-dart frog**. *Evolution* 2008, **62**(11):2742-2759.
